# Co-evolution of atypical *BRAF* and *KRAS* mutations in colorectal tumorigenesis

**DOI:** 10.1158/1541-7786.MCR-24-0464

**Published:** 2025-04-01

**Authors:** Connor E. Woolley, Enric Domingo, Juan Fernandez-Tajes, Kathryn A. F. Pennel, Patricia Roxburgh, Joanne Edwards, Susan D. Richman, Tim S. Maughan, David J. Kerr, Ignacio Soriano, Ian P. M. Tomlinson

**Affiliations:** 1 Department of Oncology, University of Oxford, Old Road Campus Research Building, Roosevelt Drive, Oxford, UK; 2 School of Cancer Science, Wolfson Wohl Cancer Research Centre, University of Glasgow, Glasgow, UK; 3 Division of Pathology and Data Analytics, University of Leeds, Leeds, UK; 4 Radcliffe Department of Medicine, University of Oxford, Oxford, UK

## Abstract

*BRAF* mutations in colorectal cancer (CRC) comprise three functional classes: Class 1 (V600E) with strong constitutive activation, Class 2 with pathogenic kinase activity lower than Class 1, and Class 3 which paradoxically lacks kinase activity. Non-Class 1 mutations associate with better prognosis, microsatellite stability, distal tumour location and better anti-EGFR response. Analysis of 13 CRC cohorts (n=6,605 tumours) compared Class 1 (n=709, 10.7% of CRCs), Class 2 (n=31, 0.47%) and Class 3 (n=81, 1.22%) mutations. Class 2- and Class 3-mutant CRCs frequently co-occurred with additional Ras pathway mutations (29.0% and 45.7% respectively vs 2.40% in Class 1, p<0.001), often at atypical sites (*KRAS* non-codon 12/13/61, *NRAS*, or *NF1*). Ras pathway activation was highest in Class 1 and lowest in Class 3, with greater distal expression of EGFR ligands (*AREG*/*EREG*) supporting weaker *BRAF* driver mutations. Unlike Class 1 mutants, Class 3 tumours resembled chromosomally-unstable CRCs in mutation burdens, signatures, driver mutations and transcriptional subtypes, while Class 2 mutants displayed intermediate characteristics. Atypical *BRAF* mutations were associated with longer overall survival than Class 1 (HR=0.25, p=0.011), but lost this advantage in cancers with additional Ras mutation (HR=0.94, p=0.86). This study supports the suggestion that Class 3 BRAF mutations amplify existing Ras signalling in a two-mutation model and that enhancement of weak/atypical Ras mutations may suffice for tumorigenesis, with potentially clinically-important heterogeneity in the Class 2/3 subgroup.

## Introduction

**Figure 1:**
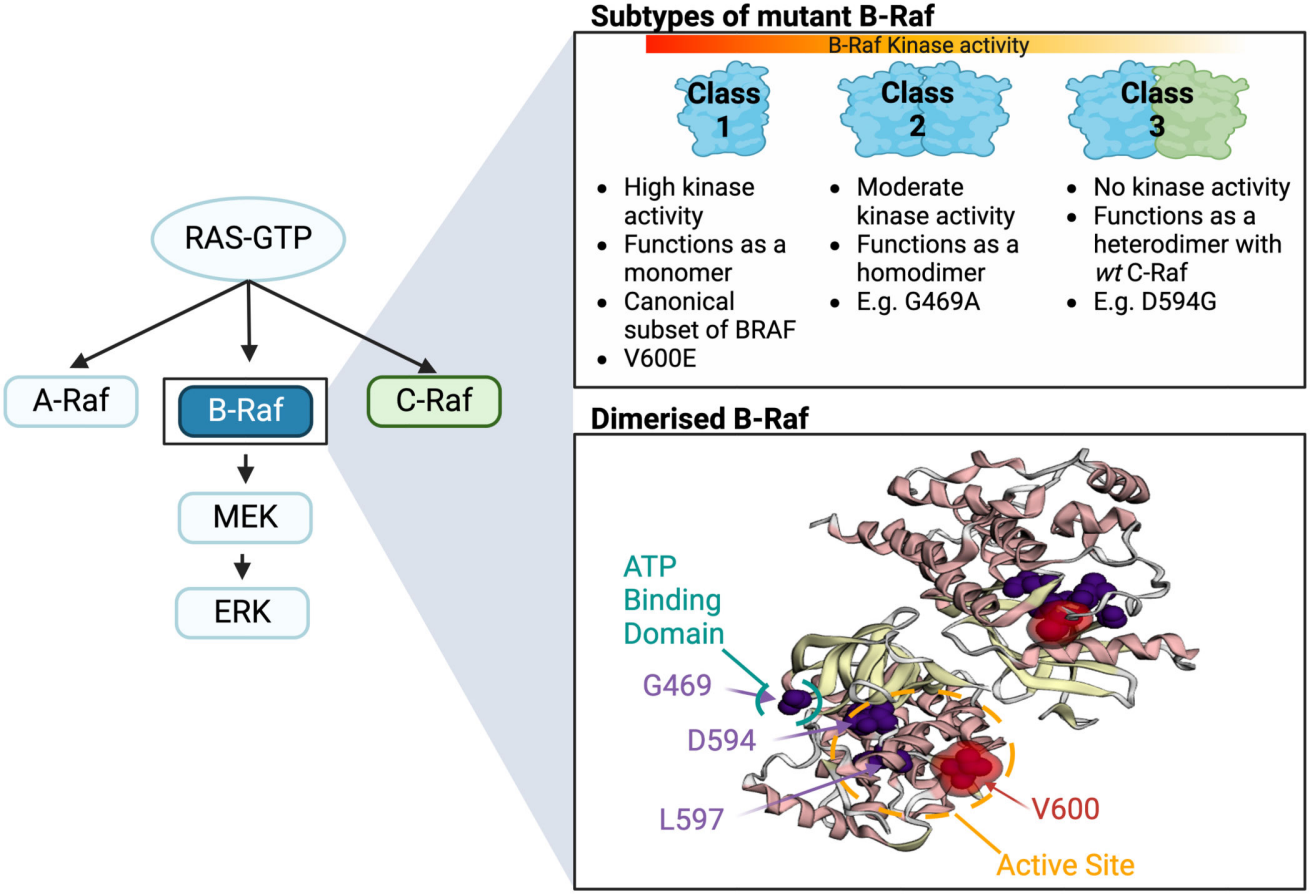
*BRAF* mutations are classified by their effects on kinase activity. B-Raf is a central component of the Ras signalling cascade, and mutations in *BRAF* are termed either Class 1, 2, or 3 based upon the resultant kinase activity. Mutations in *BRAF* are most commonly localised to the ATP binding domain and active site.

**Figure 2:**
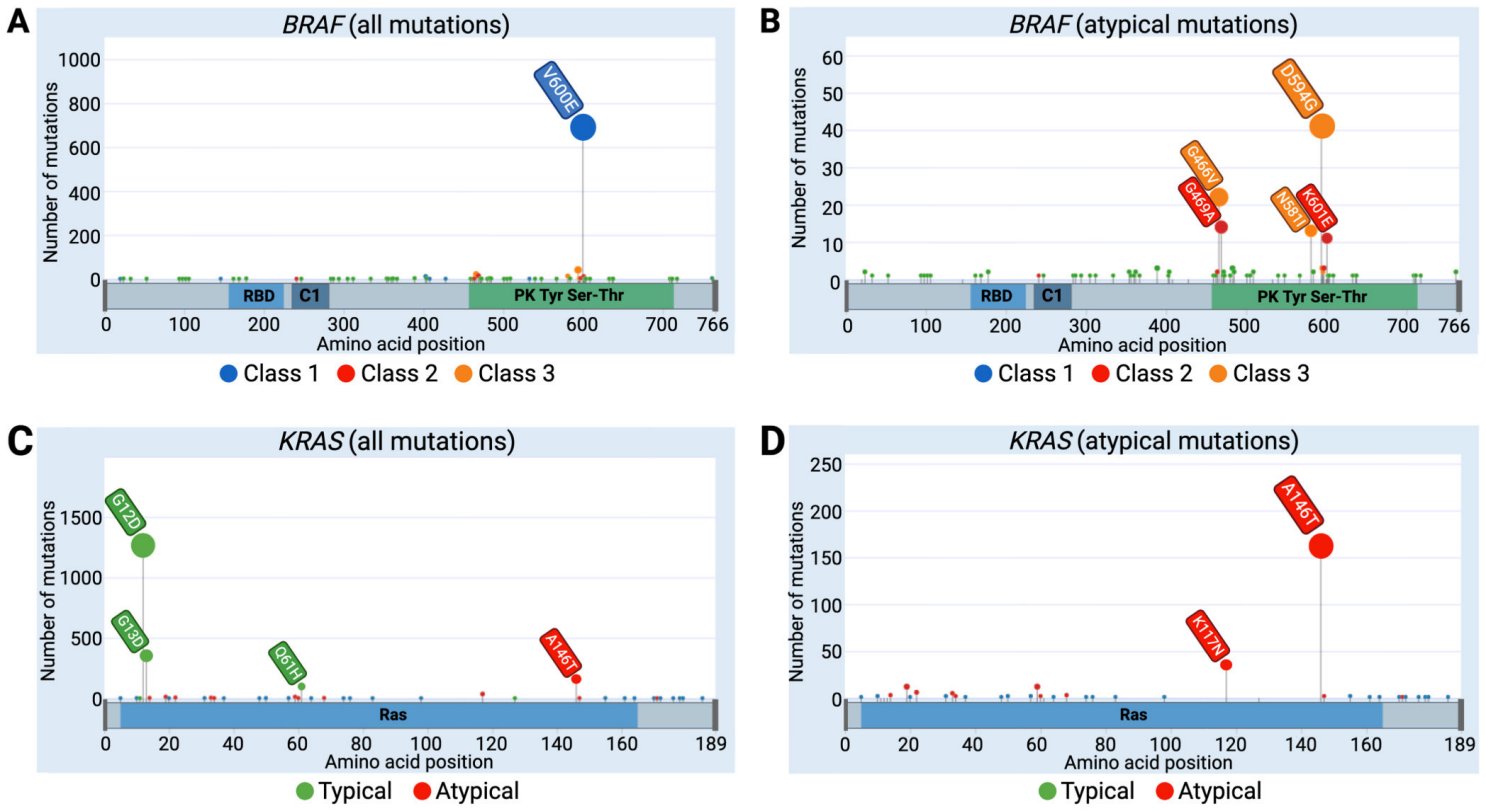
Lollipop plots representing *BRAF* and *KRAS* mutations across the combined cohorts. **A:** *BRAF* mutations showing the predominance of V600E. **B:** *BRAF* mutation distribution absent V600E shows that the second most frequent alterations are Class 3 changes (e.g. D594G and G466V). **C:** *KRAS* mutations showing predominance of ‘typical’ changes at codons 12 and 13. **D:** The distribution of *KRAS* mutations classified as ‘atypical’ in our analysis. Plots were generated using the g3viz R package (27). Note that these mutation frequencies do not necessarily correspond precisely to those in unselected CRCs owing to the inclusion criteria for several studies, especially those based on clinical trials.

Many colorectal cancers (CRCs) acquire driver mutations in genes in the MAPK/ERK signalling pathway, including heterozygous missense changes in *KRAS, NRAS* or *BRAF*. The most common *BRAF* driver mutation in CRC is a valine to glutamic acid substitution, V600E, that causes constitutive Ras pathway activation downstream of BRAF (Figure 1). V600E acts as a monomer and is termed a Class 1 mutation (1). There also exist Class 2 *BRAF* mutations (for example, at codons 469, 597, and 601) that produce active BRAF homodimers. However, an important minority of CRCs harbour Class 3 mutations in *BRAF* that paradoxically lead to a kinase-impaired protein (2), the most common being amino acid substitutions at codons 466, 594 and 596. Class 3 mutations appear to act by increasing pathway activation through enhanced stabilisation of BRAF-CRAF heterodimers in the presence of active Ras (2–4). Whilst Class 1 mutations tend to occur in CRCs of the proximal colorectum, Class 2 and 3 mutations may be over-represented in distal CRCs and might confer a relatively good prognosis (5–7). Further details of the locations of mutations of each *BRAF* class mutations are provided in the Methods and Figure 2.

The spectrum of driver mutations found in any tumour depends on both Darwinian selection (dependent on the cell’s genetic complement and tumour microenvironment) and the mutational processes active within the cell. The latter are manifest in the overall spectrum of mutations in the tumour, which can be identified through large-scale DNA sequencing. Since multiple mutational processes may act, and vary in time and space, the mutation spectrum can be deconvolved into signatures, most often based on the 96 types of base substitution in a trinucleotide context (8). Individual signatures may result from exposure to carcinogens, specific defects in DNA repair or other cellular features or processes. As a result, it is possible to infer the likelihood of a particular driver mutation arising in a cancer by comparing its trinucleotide context with the mutation spectrum and dominant mutational signatures present in that cancer. In the case of CRC, our previous work has shown that *BRAF* V600E (c.1799T>A) mutations are mutationally unlikely to occur, because they arise from a mutational channel (GTG>GAG) with low activity (9). Their high prevalence suggests that they are strongly positively selected, presumably because they efficiently activate the MAPK pathway.

The existence of Class 2 and 3 *BRAF* driver mutations, with reduced or absent kinase activity, is therefore an apparent paradox. We can envisage at least three explanations that are not mutually exclusive: (i) mutational processes strongly favour the occurrence of Class 2/3 *versus* Class 1 mutations in a minority of cancers; (ii) the differential selective advantage between Class 2/3 and Class 1 mutations is low in some microenvironments; and (iii) the relative weakness of Class 2/3 mutations is compensated by other factors, such as extra driver mutations in other genes. In this study, we use 13 CRC cohorts (Supplementary Figure 1) with genome, exome, or panel sequencing data to investigate the mutational landscape of CRCs with Class 2 or 3 *BRAF* mutations.

## Methods

### Data acquisition

Data on Ras pathway mutations in colorectal cancer were obtained from the following studies or repositories (details in Supplementary Table 1): Stratification in Colorectal Cancer (S:CORT) Consortium (10), The Cancer Genome Atlas (TCGA) (RRID:SCR_003193) (11), the Genomics England 100,000 Genomes Project (RRID:SCR_010502) (12), QUASAR 2 (13), IMAGINE (14), with publicly available cohorts accessed through the cBioPortal for cancer genomics (15) “coadread_dfci_2016”, “coadread_cptac_2019”, “coadread_casecc_2015”, “coadread Genentech”, “coadread_mskcc”, “coadread_tcga_pan_cancer”, “crc_msk_2017”, “rectal_msk_2019” and “crc_apc_impact_2020” (11,16–22). Any duplicate sample identifiers across studies were removed. All data sets had identified somatic mutations in the most frequent Ras pathway drivers in cancer, *BRAF, KRAS, NRAS* and *NF1*, by direct sequencing of all relevant exons, and many provided MSI status and routine clinicopathological data, including cancer location in the colorectum. Additional data, specifically mutations in other major and minor CRC driver genes and somatic copy number changes (SCNAs), were available from some data sets (Supplementary Table 1, Supplementary Figure 1).

### Analysis of driver status of non-V600E *BRAF* mutations using dNdScv

To investigate whether *BRAF* mutations, excluding the common V600E variant, occur under positive selection, we performed dN/dS analysis using dNdScv (RRID:SCR_001905) (23) as part of the IntOGen driver-analysis Nextflow pipeline, within the 100KGP Genomics England Research Environment. Following methods described by (24), we created separate datasets for microsatellite stable (MSS) and microsatellite instability (MSI) colorectal cancers (CRCs), with V600E variants removed from both groups. This approach allowed us to specifically examine selection pressure on non-V600E *BRAF* mutations, of which the majority in the residual cohort were missense mutations leading to Class 2 or Class 3 *BRAF*.

### Class 1, 2 and 3 *BRAF* driver mutations

Based on their over-representation above background frequencies in multiple cancer types and specific *in vitro* functional assessments in the literature (2), we refer to the following codons when classifying *BRAF* mutations:

Class 1: 600

Class 2: 464, 469, 597, and 601

Class 3: 466, 581, 594, 595, and 596

Class 2 and 3 *BRAF* mutations are collectively denoted as ‘atypical’ *BRAF* mutations below. Mutations occurring in codons other than the above were excluded from class-based analyses, as data are lacking on their functional dependence on Ras signalling and dimerisation. In exploratory analyses, we also examined *BRAF* mutations according to their locations in functional domains of the *BRAF* protein (Figure 2): A, codon 600; B, flanking codon 600 (581-601, excluding 600); and C, within the ATP binding site (464, 466, 469).

### Classification of ‘typical’ and ‘atypical’ driver mutations in *KRAS, NRAS* and *NF1*

Driver mutations in Ras pathway genes other than *BRAF* were assessed. These mutations most commonly occurred in the genes *KRAS, NRAS*, and *NF1*. Therefore, we focused upon these genes rather than other, rarely mutated genes, such as the ErbB ligands, which have the additional issue that mutation pathogenicity is often uncertain. Mutated codons in *KRAS, NRAS*, and *NF1* (collectively referred to as ‘other Ras pathway driver genes’ herein) were pre-specified as ‘typical’ or ‘atypical’ through interrogation of the COSMIC database (25), based on their over-representation above background frequencies in multiple cancer types, specific functional assessments, and frequencies in CRC. Synonymous or probably non-pathogenic mutations were not considered. For the purposes of this study, we classified *typical* pathogenic *KRAS* mutation as amino acid substitutions occurring at codons 12, 13 and 61, whereas *atypical* (yet predicted pathogenic) *KRAS* mutations were at codons 14, 19, 22, 33, 34, 59, 60, 68, 117, 146 and 147. Atypical *KRAS* mutations are often weaker drivers of Ras signalling (for example, L19F and A146T) than typical drivers (for example, G12D), however still result in pathogenic Ras pathway stimulation (26,27). All predicted pathogenic *NRAS* mutations (codons 12, 13, and 61) and *NF1* (protein-truncating) mutations were also classed as *atypical* Ras pathway mutations based on the relatively low frequency of any pathogenic mutations in these genes in CRC (28).

### Copy number analysis and clonality estimates

Statistical analysis of copy number alterations (CNA) was conducted through the *enrichments* package included in the *cBio Cancer Genomics Portal* software package, for the S:CORT and cBioPortal datasets. For 100KGP, the *Battenberg* program (RRID:SCR_017098) (29) was used to call allele-specific copy number from whole-genome sequencing data, based on cancer cell purity estimates from CCube (30). As CNA calls from Battenberg are based on germline polymorphisms, they do not attribute copy number to the mutant or wildtype allele at oncogenes such as *BRAF, KRAS* or *NRAS*. An expression (Eq. 1) was therefore derived to assign the copy number of each oncogene allele. If *VAFMut* is the frequency of the mutant allele in the cancer sequencing reads, *Nmut* is the putative copy number of the mutant allele, *Nwtcan* is the copy number of wildtype allele, *2* represents the number of copies of the wildtype allele in a normal cell, and Purity represents the calculated tumour purity (proportion of cancer cells in the sample), substitution of each allele-specific copy number value into Eq. 1 as either *Nmut* or *Nwtcan* in turn generally provides only one valid solution to the CCube-estimated tumour purity, thus allowing assignment of *Nmut* and *Nwtcan*.

Eq. 1 $$\;Purity\;=\frac{2}{\left(\frac{Nmut}{\;VAFMut\;}\right)-Nmut-Nwtcan+2}$$

### Assessing mutation clonality in tumours

For patients where archival FFPE tissues were available, three 10 μm sections were micro-dissected with a needle into multiple regions for extraction and processed for DNA using the Roche High Pure FFPET DNA Kit using the manufacturer recommended protocol. Purified DNA was eluted into 50μl and target exons for *BRAF* and concomitant Ras (*KRAS*/*NRAS*) were amplified with high fidelity polymerase (Q5 Hot Start HiFi Polymerase, NEB). Primers for amplification can be found in Supplementary Table 2. Annealing temperatures were determined via the NEB TM Calculator (https://tmcalculator.neb.com/!/main) and an extension time of 30 seconds. PCR amplicons were purified using the Qiagen QIAquick PCR Purification Kit and eluted into 30 μl prior to submission for Sanger sequencing using the same forward and reverse primer pairs for initial amplification. Sequencing chromatograms were visualised in Snapgene.

### Mutation spectra and signatures

The *SigProfilerExtractor* package (RRID:SCR_023121) (31) was used to produce genome-wide 96-channel mutation spectra for WGS samples in the 100KGP dataset. Mutations are presented in a trinucleotide (3nt) context, wherein each 3nt context represents a mutational channel, and reported from the pyrimidine strand for conciseness. With raw 96-channel counts called for each sample, they were normalised to proportional activity within the sample and thus summed to 1, allowing comparison between samples. The mutation spectrum for each sample was fitted against COSMIC SBS signatures (version 3) (32) to deconvolve the contributing signatures and thus infer the mutational processes undergone by the tumour throughout its lifetime.

### Logistic regression analysis of mutation channel activities

Logistic regression was performed in R (version 4.2.1) (RRID:SCR_001905) using the base *glm* package, with models incorporating the explanatory variables ‘Casual channel proportion’ or ‘V600E channel proportion’. Causal/V600E channel proportions were calculated as the proportion of trinucleotide (3nt) channel mutations, out of all 3nt channel mutations within the sample, responsible for generating the *BRAF* mutation. Covariates included in all models were tumour location (as either proximal or distal colorectum), MSI status, age at diagnosis, and patient sex.

### Regional expression analysis of Ras/EGFR pathway genes in normal colorectal tissues

Differential gene expression analysis across core Ras/Raf pathway genes *KRAS, BRAF, and NRAS*, plus the EGFR ligand genes *AREG* and *EREG*, was conducted across normal RNA-seq samples from Fernandez-Rozadilla *et al*. (33) and subsequently validated using normal samples present in The Cancer Genome Atlas (TCGA) COAD-READ cohorts (11). Expression analysis was conducted in R (4.2.1) using DESeq2 (1.4.2) (RRID:SCR_015687) (34) with tissue location as the condition variable. Only samples with specific anatomical location available were included, with distal colon and rectum merged to create a distal/rectal grouping for contrast against the proximal colon. Multiple-testing corrections were performed using the Benjamini-Hochberg procedure.

### Gene set enrichment analysis

The GSEA tool (version 4.1.0) (RRID:SCR_003199) was obtained from the Broad Institute’s GSEA portal (http://www.gsea-msigdb.org/gsea/index.jsp) and gene signatures extracted from MSigDb (http://www.gsea-msigdb.org/gsea/msigdb/index.jsp) (RRID:SCR_016863). Analysis made use of the curated oncogenic pathways (C6) with 10,000 permutations. All other parameters were set to their default values, with results considered significant at an FDR adjusted q-value of 0.05 and a normalised enrichment score (NES) of < -1.2 or >1.2 depending upon signature directionality.

### Consensus Molecular Subtypes (CMS) by *BRAF* class

**Figure 3:**
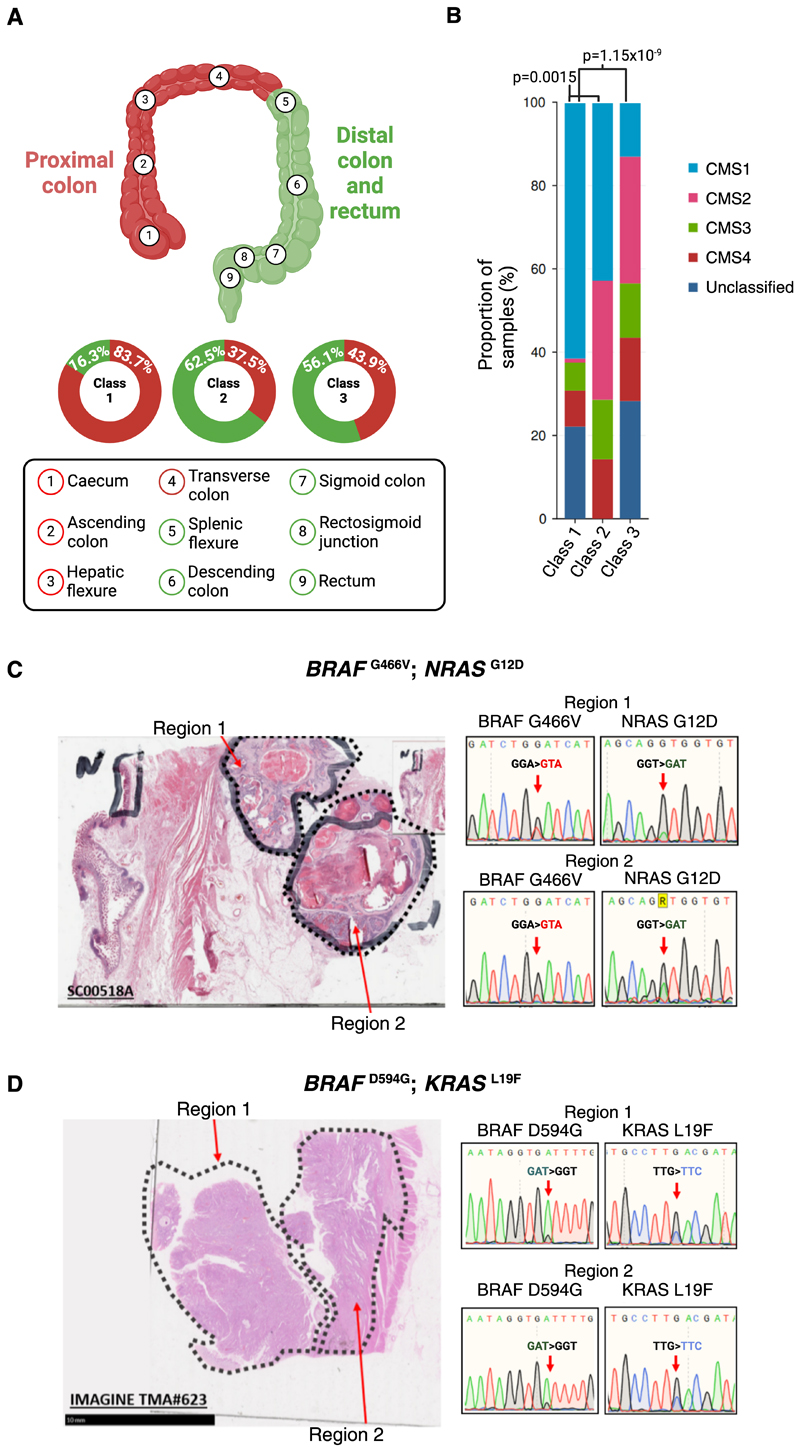
Classes of *BRAF* mutation exhibit distinct clinicopathological and molecular characteristics. **A:** Sub-division of the large bowel into proximal and distal colorectum. Proportions of CRCs in each location are shown by *BRAF* mutation class. **B:** Consensus molecular subtype (CMS) classifications of *BRAF*-mutant CRCs by mutation class. The differences between Class 2 or 3 versus Class 1 (V600E) were highly significant (p<0.001). No significant difference was observed between Classes 2 and 3 (p=0.270). **C-D:** Sanger sequencing chromatograms highlighting the spatial co-occurrence of *BRAF* and additional Ras pathway mutations in two CRCs. Two regions of each cancer were microdissected, DNA was extracted, and relevant exons were PCR-amplified prior to Sanger sequencing. Illustrative results are shown. The co-occurrence of *BRAF* G466V and *NRAS* G12D can be observed in tumour 1 (C), and of *BRAF* D594G and *KRAS* L19F in tumour 2 (D). This analysis does not exclude fine-scale spatial mixing of distinct sub-clones but is consistent with the two mutations tested being present in the same tumour cells.

CMS subtypes were called as part of the S:CORT consortium workflow, using transcriptomic array data generated via Almac XCel array. Initial comparisons between Classes as shown in Figure 3B were performed by Fisher’s exact tests. Multiple logistic regression models controlling for covariates were produced in R using the base *glm* package, with each CMS group used as the outcome variable. *BRAF* class and CMS groups were treated as categorical variables and dummy coded, with each CMS group modelled per *BRAF* class. Covariates included in all models were age at diagnosis, tumour location (proximal or distal colorectum), MSI status, and stage.

### Survival analysis

Statistical analyses based on survival data were performed in R (version 4.2.1) (35) using mixed-effects Cox proportional hazards models through the *coxme, survminer* (RRID:SCR_021904), and *survival* packages. Cohort studies used for survival analysis, with overall survival (OS) data available were: 100,000 Genomes Project CRC; S:CORT; TCGA COADREAD; CPTAC 2019; CRC MSK 2017; and CRC APC IMPACT 2020. For all mixed-effects Cox models models, the following were included as fixed-effects covariates: age at diagnosis, patient sex, tumour location (left/right), and cancer stage. Study cohort was included in all models as a random-effect covariate. Proportional hazards assumptions were tested using scaled Schoenfeld residuals from corresponding fixed-effects models. In Model I (Survival by *BRAF* class irrespective of additional Ras), Stage showed a minor violation (p=0.017), driven by sparse events at late follow-up (Supplementary Figure 2A). Given Stage’s clinical importance and stable effect during the majority of follow-up, we retained it in the final models, interpreting its effect as an average over time. No violation of proportional hazards was observed for Stage in Model II (p=0.109) (Supplementary Figure 2B), while Model III (the effect of additional Ras status within Class 3 *BRAF* mutant CRCs) showed a stronger Stage violation (p=0.001, Supplementary Figure 2C) due to limited sample size in the Class 3-only cohort, precluding interpreting of Stage effects, the main variable of interest (additional Ras status) met proportional hazards assumptions (p=0.18), allowing valid interpretation of these results.

## Results

### Class 2 and 3 *BRAF* mutations show positive selection in CRC and frequently co-occur with other Ras pathway mutations

To explore the driver status of non-V600E *BRAF* mutations in CRC, particularly Classes 2 and 3, we employed dNdScv analysis (23), which identifies genes under significant selection pressure within cancer cohorts. A dN/dS ratio exceeding one indicates positive selection of non-synonymous mutations, suggesting potential driver status. Using the 100KGP whole-genome sequencing cohort, we analysed MSS and MSI primary CRCs separately (as described in Cornish *et al*. (24)after excluding V600E variants. The remaining non-V600E *BRAF* variants predominantly comprised missense substitutions resulting in Class 2 or Class 3 mutations. In MSS primary tumours, non-V600E BRAF missense mutations showed significant positive selection (dN/dS = 5.43, q=0.012). In contrast, MSI tumours showed no significant positive selection for non-V600E BRAF mutations (missense dN/dS = 1.14, q=0.99). Separate analyses of Class 2 and Class 3 mutations independently showed nominally significant positive selection of *BRAF* in MSS tumours for both classes (dN/dS=7.21, p=0.013 and dN/dS=8.51, p=0.005 respectively).

The *KRAS* and *BRAF* mutations found in the combined cohorts are shown in Figure 2. Compared with tumours harbouring Class 1 mutations, CRCs with atypical *BRAF* mutations in Classes 2 and 3 tended to occur in the distal large bowel (Table 1, Figure 3A), consistent with previous reports (5,7,36) (58% vs 16.3%, distal v proximal colorectum) (p<0.001, Fisher’s exact test). There was no significant difference in location between Classes 2 and 3 (62.5% vs 56.1% in distal colorectum, p=1.0, Fisher’s exact test). Further exploration of the Class 3 cohort alone showed that mutations at codon 466 were strongly associated with proximal location, in contrast to other Class 3 variants (13/18 *versus* 12/39 respectively, p=0.005, Fisher’s exact test). No significant differences were observed between age of sampling and *BRAF* mutation class.

The overall frequency of Class 1 *BRAF* mutations, all bar one of which were V600E, in our combined data set was 10.7% (709/6605). Values per cohort are presented in Supplementary Table 3a. Largely as expected, V600E alterations were almost entirely mutually exclusive with other Ras pathway driver mutations (37–39). Seventeen CRCs harbouring a V600E mutation also presented with additional, predicted pathogenic mutations in *KRAS, NRAS*, or *NF1* (17/709, 2.40%), comprising 13 with *NF1* inactivating mutations and four with *KRAS* mutations (4/709, 0.71%, mostly G12D).

We then assessed the 81 (81/6605, 1.22%) CRCs with a Class 3 *BRAF* mutation (codons 466, 581, 594, 595, and 596). Thirty-seven (37/81, 45.7%) of these tumours additionally carried a pathogenic mutation in *KRAS, NRAS*, and/or *NF1* (Table 1). One Class 3 mutant tumour had an additional *BRAF* mutation, harbouring both D594G and a Class 2 change, G469V. Of the 31 (31/6605, 0.47%) CRCs with Class 2 *BRAF* mutations (codons 464, 469, 597, and 601), nine had additional pathogenic Ras pathway mutations (9/31, 29.0%). The over-representation of additional Ras pathway driver mutations in CRCs with Class 3 *BRAF* mutations was very strong relative to Class 1 *BRAF* mutant CRCs (45.7% vs 2.40%, P<0.001, Fisher’s exact test), and a slightly weaker association was present in Class 2-mutant tumours relative to Class 1 (29.0% vs 2.40%, p<0.001, Fisher’s exact test).

Focussing on the 37 CRCs with Class 3 *BRAF* mutants and concomitant Ras pathway mutations, only 8 harboured typical *KRAS* mutations (p<10^-5^, Fisher’s exact test). Notable atypical Ras pathway mutations in the 29 other tumours included 18 changes in *KRAS* (including V14I, L19F, P34L, G60D, K117Q and A146V) and 12 in *NRAS*. One cancer had a Class 3 mutation (G466V), two atypical *KRAS* mutations (V14L;D33E) and an *NRAS* mutation (G12V). Six of the nine additional Ras mutations in Class 2 tumours were also atypical, comprising four *KRAS* changes (A59T, A146V, A146T x 2) and two *NRAS* mutations (G12D and G12V). The frequency of atypical Ras pathway mutations in particular was higher than expected overall for Class 2 and 3 *BRAF-*mutant cancers when compared to Class 1 cancers (13/709 30.6% versus 34/112 1.8% respectively, p<0.001, Fisher’s exact test) (Table 1). There was also a borderline significant difference observed between Class 2 and Class 3 *BRAF* mutant CRCs (frequencies 16.1% and 35.8% respectively; p=0.06, Fisher’s exact test) (Table 1).

**Figure 4:**
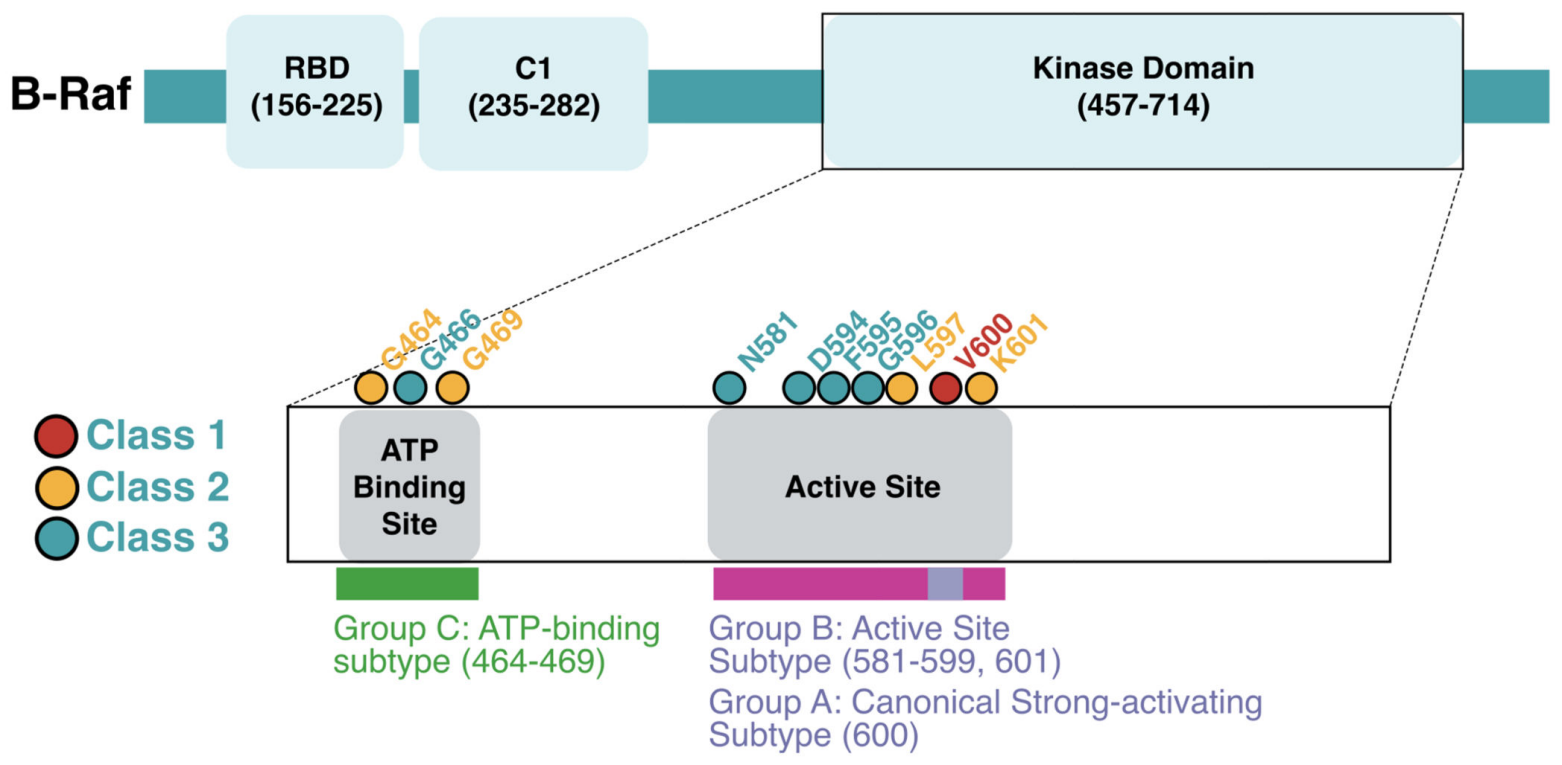
*BRAF* functional domains and comparison between the 1-2-3 and A-B-C *BRAF* classification systems. The 1-2-3 *BRAF* mutation classification is based on *in vitro* assays of Ras pathway activity. However, as it has not been validated by analysis of native mutations in isogenic cells, we explored, in parallel, a simpler classification based on mutation location in functional domains: A, codon 600; B, other active site mutations flanking codon 600 (581-601); and C, ATP binding site mutations (464-469). RBD=Ras Binding Domain.

The 1-2-3 *BRAF* mutation classification is based on *in vitro* assays of Ras pathway activity, although these were not native mutations in isogenic cells. We therefore considered, in parallel, a simpler classification based on mutation location in functional domains: A, codon 600; B, flanking codon 600 (581-601, excluding 600); and C, within the ATP binding site (464, 466, 469) (Supplementary Tables 3b and 4, Figure 4). In agreement with the conventional classification, we found strongly significant associations between Classes B and C and the presence of additional Ras pathway mutations, in comparison to class A which mostly comprises V600E (2.40% for class A versus 35.6% and 52.5% for B and C respectively, p<0.001, Fisher’s exact tests) (Supplementary Table 4). Additional analyses using the exploratory A-B-C classification are presented in Supplementary Text 1.

### Clonality of atypical BRAF and additional Ras mutations

In Genomics England 100,000 Genomes Project CRCs with either a Class 2 or 3 *BRAF* variant and an additional Ras pathway variant, we investigated whether these mutations were present in different cancer sub-clones or in the same cell. By integrating the estimated purity of each tumour, the observed allele frequency of each mutation of interest, and allele-specific copy numbers, we estimated a corrected allele frequency for the *BRAF* variant and the other Ras variant (details in Methods *Copy Number Analysis*). We then used these estimates to correct mutant and reference read counts to the underlying copy number and tested whether the counts of the two variants differed significantly from the estimated tumour purity at nominal *p<*0.05, thus suggesting whether they are clonal or subclonal, whether nested or entirely distinct. In 7/8 cancers analysed, the data were consistent with atypical *BRAF* and concomitant Ras pathway mutations being present in the same clonal population (Chi-square tests of independence, Supplementary Table 5).

We further obtained archival tumour material from two *BRAF*-Ras double-mutant CRCs (Figure 3C-D). H&E sections were dissected into multiple, spatially distinct regions within each tumour and directly Sanger sequenced for the relevant region of *BRAF* and either *KRAS* or *NRAS* as appropriate. Sanger sequencing chromatograms sampled in Figure 3 are provided in an accompanying zip archive as Supplementary Data 1. In both cases, there was concordance between the two mutations. We thus concluded that, in general, atypical *BRAF* mutations and concomitant additional Ras pathway mutations are likely to be present in the same cancer cells.

We also explored whether gene amplification was used by tumours to boost the putative weak effects of atypical *BRAF* mutations, as an alternative to acquiring atypical Ras mutations. For samples harbouring Class 2 or 3 *BRAF* mutations, we identified no association between absence of an additional Ras pathway mutation and presence of a *BRAF* amplification (copy number greater than 2, p=1, Fisher’s exact test). We further found no significant differences in gene amplification when stratifying Class 2 and Class 3 *BRAF* mutants by presence or absence of additional pathogenic mutation in *KRAS, NRAS*, or *NF1* (p>0.05). These data suggest that even Class 2 atypical *BRAF* mutations do not have strong enough intrinsic Ras activity to be boosted to pathogenic levels by increased copy number.

### Atypical *BRAF* mutations and additional Ras pathway mutations cannot be explained by mutator phenotypes

Two main acquired mutator phenotypes are well described in CRCs, microsatellite instability (MSI) caused by defective DNA mismatch repair and aberrant polymerase proofreading caused by *POLE* mutations. Both of these cancer types have a large excess of base substitutions. Based on previous models of ‘polygenic’ tumorigenesis in hypermutant cancers (40), we wondered whether the multiple *BRAF* and Ras pathway mutations in Class 2 and 3 *BRAF-*mutant CRCs were driven by a mutator phenotype. Contrary to this hypothesis, in samples with available MSI data (Table 1), we found an over-representation of MSI in Class 1-mutant tumours – reflecting the well-established MSI-V600E association (41) – with Class 2 and 3 tumours all MSI- (52.4% v 0%, p<0.001, Fisher’s exact test). None of the Class 2 and 3 *BRAF*-mutant CRCs had pathogenic *POLE* mutations.

Class 1 *BRAF* mutations are also associated with the CpG Island Methylator Phenotype (CIMP) and with Wnt pathway activation *via RNF43* or *CTNNB1* mutations. Within the S:CORT dataset, Class 1 CRCs were predominantly CIMP-H (71/118, 60.2%), compared to only 26.7% (8/30, 26.7%) in Class 3, p=0.002, Fisher’s exact test). The largest CIMP cluster for Class 3 mutant CRCs was CIMP-L at 40% (12/31), not significantly different from Class 1 (27.1%, 32/118; p=0.30).

Relatedly, 20.1% (133/661) of tumours with available data harboured a pathogenic *APC* mutation (11), whereas 81.4% (22/27) of Class 2 and 76.7% (56/73) of Class 3 respectively did so (p<0.001, Fisher’s exact tests.). We further found significantly lower frequencies of pathogenic *CTNNB1* mutation in Classes 2 and 3 tumours versus Class 1 (1.02%, 1/98 vs 16.7%, 96/575, p<0.001, Fisher’s exact test), along with reduced frequencies of pathogenic *RNF43* mutation for Class 2 and 3 versus Class 1 (0% 0/98 vs 22.9% 147/643, p<0.001, Fisher’s exact test). Thus, atypical *BRAF* mutations appear not to follow the serrated/MSI/CIMP/RNF43 pathway of colorectal tumorigenesis, but instead resemble cancers following the canonical pathway of colorectal tumorigenesis (11,42).

### *BRAF* V600E mutations are under strong selective constraints, whereas atypical *BRAF* variants are subject to weaker constraints

Although we found no evidence that atypical *BRAF* mutations are associated with the MSI or *POLE* mutator phenotypes, it remained possible that their occurrence was driven by other mutational processes that are active in tumour cells or their precursors, mostly in the distal colorectum. We therefore generated whole-genome 96-channel (trinucleotide context) mutational spectra for each of the 100KGP Genomics England tumours (WGS) data. The mean proportional activity of the Class 1 (V600E) mutation channel, GTG>GAG, was 0.69%, compared to a summed average of 12.9% for the 16 Class 2 and 3 mutation channels. Presence of a V600E mutation was not associated with activity of the causal channel (p=0.286, logistic regression), whereas presence of an atypical *BRAF* mutation was associated with higher activity of the causal channels (p=0.028) (Supplementary Table 6). These data are consistent with V600E being under strong selective constraints in the proximal large bowel whereas atypical *BRAF* mutations have weaker constraints and are influenced more by mutational processes.

### The consensus molecular subtypes (CMS) of Class 3 mutant *BRAF* CRCs differ from those of Class 1 mutants

The consensus molecular subtypes (CMS) of CRC (43) represent a gene expression-based stratification into four subtypes. CMS1 (referred to as the *MSI Immune* subtype) is the predominant signature associated with Class 1 *BRAF* mutations, and is characterised by MSI, high CIMP, and hypermutation. Based on S:CORT data, we found a significant difference in CMS classification across all three *BRAF* mutation classes. CMS data were available for 114 Class 1, four Class 2, and 28 Class 3 *BRAF* mutant cancers. CMS1 predominated in Class 1-mutant cancers, as expected (44). However, Class 2 and Class 3 tumours had a lower proportion of CMS1 and higher proportion of CMS2 (Figure 3B; p<0.001, Fisher’s exact tests). CMS3 and CMS4 occurred at a similarly low frequencies in the three classes. CMS2 is generally regarded as the *canonical* CMS subtype, characterised by high levels of somatic copy number alterations and increased of Wnt and Myc signalling. To ensure the apparent differences in CMS between *BRAF* mutation classes were not readily explained by potentially confounding variables including MSI status and tumour location (see Methods), we further performed multiple logistic regression analyses (Supplementary Table 7). *BRAF* Class 1 mutant CRCs were significantly more likely to be CMS1 (OR=6.68, p=0.024), and significantly less likely to be CMS2 (OR=0.01, p=0.01) than cancers in the other two *BRAF* classes. Class 3 mutant CRCs showed the opposite associations to Class 1 and were significantly less likely to be CMS1 (OR=0.15, p=0.05), and highly likely to be CMS2 (OR=33.04, p=0.004). Class 2 mutant CRCs were not associated with any significant differences in CMS versus non-Class 2 *BRAF*, representing an intermediate subgroup between Classes 1 and 3. We additionally subtyped BRAF mutant CRCs using our own A-B-C positional classifier with consideration for additional Ras status (Supplementary Figure 3).

### Greater EGFR ligand (*AREG/EREG*) expression in the distal colorectum may support a permissive environment for atypical *BRAF* mutations

*In vitro* evidence suggests that atypical *BRAF* mutations more weakly activate Ras/Raf-MEK-ERK signalling (2,3). We hypothesised that although additional Ras pathway mutations are also likely to be weaker in their effects, they may be compensated for by another unknown source, for instance there may exist a more permissive environment in the normal distal colorectum relative to the proximal colorectum, which might permit the emergence of weaker drivers such as Class 3 *BRAF* mutations. Using normal bowel mRNA sequencing data collected as part of the Fernandez-Rozadilla *et al*. (33), subsequently referred to as “INTERMPHEN”, we first examined the expression of core Ras pathway genes *KRAS, BRAF*, and *NRAS* in samples collected from the proximal colorectum (n=119) and two sites in the distal colorectum (distal colon and rectum, n=236). *KRAS* and *BRAF* pathway genes showed only modest differences in expression among site (*KRAS* Log_2_FC=0.12, *BRAF* Log_2_FC=-0.13, proximal vs distal/rectal, p.adj<0.001), while *NRAS* expression was more greatly under expressed in distal/rectal versus proximal (Log_2_FC=-0.35, p.adj<0.001). Replication across TCGA-COADREAD normal tissue mRNA-seq (n=18 proximal, n=19 distal/rectal), however, found no significant differences in *KRAS, BRAF, or NRAS* expression (Supplementary Table 8).

**Figure 5:**
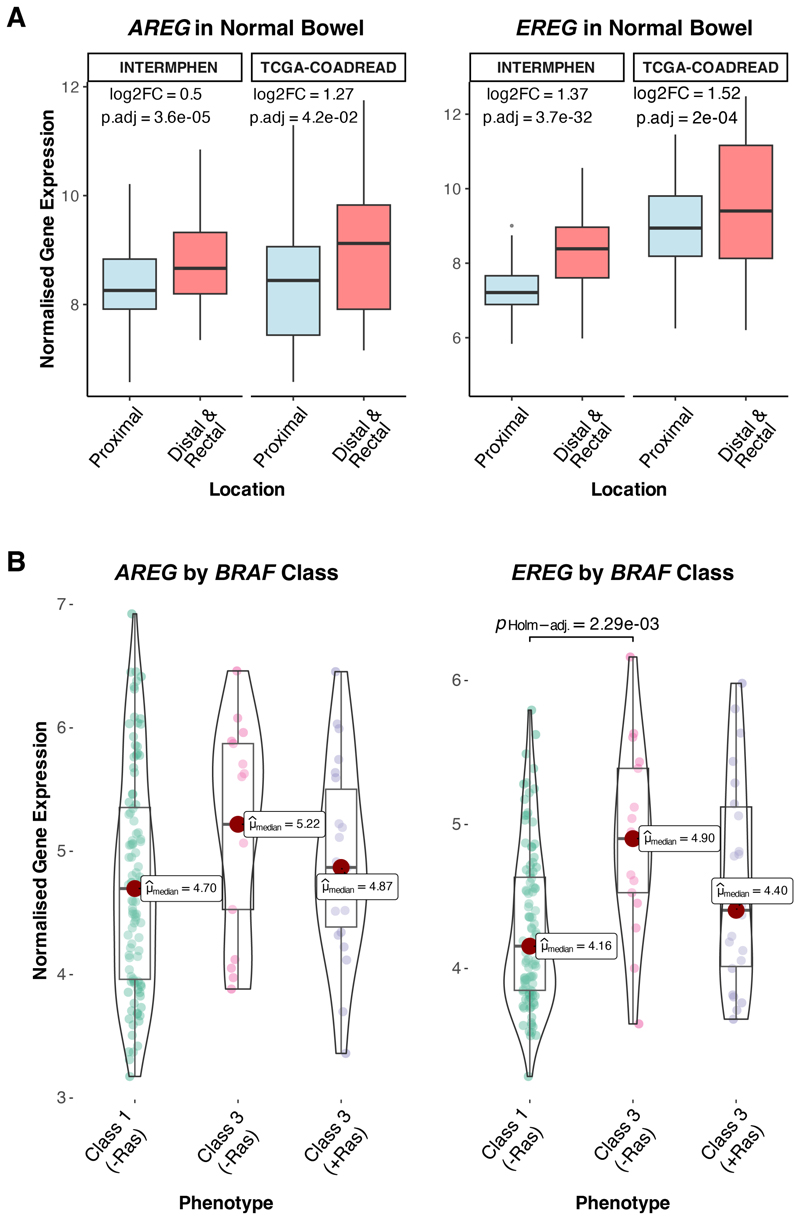
mRNA expression of the epidermal growth factor receptor (EGFR) ligands amphiregulin (*AREG*) and epiregulin (*EREG*). **A:** Expression of *AREG* and *EREG* is significantly increased in the distal colorectum versus proximal colon across the INTERMPHEN (n=119 proximal, n=236 distal colorectum) and TCGA-COADREAD (n=18 proximal, n=19 distal/rectal). Log_2_FC and significance values obtained via DESeq2 analysis (see Methods). **B:** Expression of *EREG* is significantly increased in Class 3 *BRAF*-mutant CRCs versus Class 1 in the absence of a concomitant Ras mutation (p.adj<0.005), which may reflect the distal colorectum bias of Class 3 mutations.

Notably, we found that the epidermal growth factor receptor (EGFR) ligands amphiregulin (*AREG*) or epiregulin (*EREG*), which may stimulate the Ras/Raf-MEK-ERK pathway via ligand drive (45,46), showed significant regional differences in both the INTERMPHEN and TCGA normal cohorts. In the INTERMPHEN cohort, *EREG* expression was particularly elevated in the distal colon and rectum versus proximal colon (Log_2_FC=1.37, p.adj<0.001), with *AREG* showing a similar but more modest pattern (Log_2_FC=0.50, p.adj<0.001); this finding was consistent following independent replication in the TCGA-COADREAD normal mRNA-seq cohort, with distal/rectal enrichment of *EREG* (Log_2_FC=1.52, p.adj<0.01) and *AREG* (Log_2_FC=1.27, p.adj<0.05) versus proximal colon (Supplementary Table 8, Figure 5A). Consistent in these regional differences in normal tissues, analysis of the S:CORT cohort, which contained sufficient atypical CRCs with RNA data, showed significantly higher expression of *EREG* in Class 3 *BRAF* mutant tumours lacking additional Ras mutation compared to Class 1 mutants (p<0.005, Dunn’s test) (Figure 5B). While this difference in *EREG* expression between *BRAF* classes may partially reflect their distinct anatomical distributions, the observation that Class 3 *BRAF* mutants with additional Ras pathway mutations showed similar EGFR ligand expression to Class 1 mutants suggests that elevated *EGFR* ligand expression might not be necessary when additional Ras pathway mutations are present. These findings suggest a natural gradient of EGFR ligand expression along the colorectum which may indeed contribute to the regional distribution of *BRAF* mutation classes, potentially providing a more supportive environment for weaker oncogenic drivers in the distal colon and rectum.

### Raf signalling levels are highest in Class 1 *BRAF* mutant cancers, while Class 3 *BRAF* mutants increase Raf signalling by additional Ras pathway mutations

To explore the *BRAF* mutant transcriptome further and to identify other differences between tumours with typical and atypical *BRAF*, we performed a hypothesis-free search for differential gene set expression between Class 3- and Class 1-mutant tumours from S:CORT, using gene set enrichment analysis (GSEA) based on C6 oncogenic gene sets. As a baseline, we first compared Class 3 mutants to Class 1, in both cases without additional Ras mutations. Of nine significantly enriched (q<0.05) gene sets transcriptome-wide (Supplementary Table 9a), at least three were clearly associated with higher Ras/Raf-MEK-ERK signalling in the Class 1 tumours: *ERRB2_UP.V1* (q=0.005); *MEK_UP.V1* (q=0.007); and *EGFR_UP.V1* (q=0.032). Furthermore, another gene set related to Wnt, decreased *MYC* signalling, was lower in Class 1 than Class 3 (MYC_UP.V1_DN, q=0.042). Comparisons between Class 1 (lacking extra Ras) and Class 3 CRCs with additional Ras (either typical or atypical) appeared to eliminate significant differences between subtypes, with none of the above gene sets emerging at q<0.05 (Supplementary Table 9b). Comparing Class 3 *BRAF* lacking additional Ras against Class 3 with typical Ras mutations showed higher SINGH_KRAS_DEPENDENCY_SIGNATURE (q=0.032) and *ERBB2_UP.V1* gene set expression (q=0.043) (Supplementary Table 9c) in the latter. However, comparison of Class 3 *BRAF* lacking additional Ras against Class 3 with additional atypical Ras yielded no significant gene sets at q<0.05 (Supplementary Table 9d). Finally, no significant differences were found between the relatively few Class 3 harbouring atypical and typical Ras (Supplementary Table 9e).

### Associations between atypical *BRAF* mutations and prognosis

Previous work has proposed that non-V600E *BRAF-*mutant CRCs have a relatively good prognosis (5–7,36), although not all such analyses have controlled for potential confounders such as MSI and tumour location, or taken into account additional Ras pathway mutations. We therefore investigated overall survival (OS) in the 6 cohorts with available data (Supplementary Data). An initial uncorrected log-rank analysis showed a borderline significantly better OS of Class 3 *BRAF* mutations versus Class 1 if both lacked additional Ras, in agreement with previous studies (p=0.058 log-rank test, Supplementary Figure 4A).

Owing to the combination of multiple patient cohorts in our dataset and the strong possibility of confounders, we proceeded to a more robust approach utilising mixed-effects Cox proportional hazards models, wherein study was treated as a random variable, while patient characteristics were treated as fixed effects variables (see Methods). Initially, we confirmed longer OS for Class 3 than Class 1 *BRAF*-mutant CRCs without concomitant pathogenic Ras pathway mutation (HR=0.25, p=0.011). No significant difference in survival was observed for Class 2 *BRAF* mutations relative to Class 1 (HR=1.13, p=0.85; Table 2, Model I).

We subsequently explored whether Class 3 cancers harbouring additional Ras mutations had a significantly different prognosis from that of Class 1 (all V600E here). No OS difference was detected between the two groups (Class 3 plus Ras HR=0.93, p=0.86; Table 2, Model II). Since concomitant Ras pathway mutations appeared to make the survival of Class 3 CRC patients similar to that of Class 1, we compared the OS of Class 3 cancers with and without additional Ras pathway mutations. We detected significantly worse OS of the tumours with additional Ras mutations (HR=4.92, p=0.03; Table 2, Model III). Overall, our results suggest that *BRAF* Class 3 CRCs have better survival than Class 1 CRCs, but the additional Ras pathway mutations in some Class 3 BRAF mutant CRCs cause survival similar to that associated with Class 1 V600E. For visualisation purposes, unadjusted Kaplan-Meier curves representing the above survival comparisons are presented in Supplementary Figure 4, while statistical results are derived from the mixed-effects models that account for potential confounders and study-specific effects.

### Are atypical *KRAS* mutations associated with additional Ras pathway mutations?

By analogy with *BRAF* mutations, we wondered whether atypical *KRAS* mutations in colorectal cancers (CRCs) without *BRAF* mutations also tended to have concomitant mutations in Ras pathway genes (Supplementary Figure 5). Of 335 CRCs harbouring atypical *KRAS* mutations in the combined cohorts, 317 (94.6%) had no concomitant pathogenic *BRAF* mutation, with *BRAF* mutations in the remaining 18 all belonging to Class 3 (18/335, 5.4%). Out of these 317 cases, 13 (4.1%) harboured an additional pathogenic Ras pathway mutation (analysis restricted to *NRAS* and *NF1*). 13 of the 335 (3.88%) CRCs with atypical *KRAS* also carried a typical *KRAS* mutation, and none of those tumours had an additional *BRAF, NRAS*, or *NF1* mutation. Most cancers with atypical *KRAS* mutations (291/335, 86.9%) had no additional mutations in *BRAF, NRAS, or NF1*, far lower than the incidence of concomitant Ras in atypical *BRAF* CRCs (Table 1). It appears, therefore, that atypical *KRAS* mutations may generally cause sufficient Ras dysregulation to drive tumorigenesis.

## Discussion

We set out to obtain clues as to why some CRCs grow with atypical *BRAF* driver mutations that are predicted to have impaired or sub-optimal kinase activity, and thus result in weak downstream MAPK signalling in comparison with the most frequently observed V600E Class 1 variant. We have shown that atypical (Class 2 or 3) *BRAF* mutations are associated with the presence of additional mutations in the Ras pathway. These frequently take the form of pathogenic changes at sites other than the *KRAS* codon 12, 13 and 61 hotspots. The additional Ras mutations probably have relatively weak effects on Ras activation (47) and seem to be present in the same cells as the atypical *BRAF* changes, rather than in a separate sub-clone. These associations appear stronger for Class 3 (loss-of-function) mutations than for Class 2 (active dimer) mutations, consistent with a model in which kinase-dead Class 3 *BRAF* mutants require concomitant Ras activation to be pathogenic (3). In Class 3 cancers that do not have additional Ras pathway mutations, this model predicts that other sources of Ras activation may be present. In support of the model, we found evidence of higher EGFR ligand expression in Class 3 than Class 1 tumours, although we cannot exclude the possibility that this results from the effects of feedback inhibition. Importantly, in normal bowel RNA-seq data, we identified increased basal *AREG* and *EREG* expression in the distal colorectum versus proximal. This regional variation in EGFR signalling may contribute to the observed Class 3 versus Class 1 differences and could explain why the distal colorectum provides a more permissive environment for atypical *BRAF* mutations to drive tumorigenesis. However, we note that transcriptomic data alone cannot fully capture pathway regulation at the protein level.

Class 2 mutations appear to be intermediate between Classes 1 and 3 in most respects. For example, CRCs with Class 1 *BRAF* mutations are likely to be CMS group 1, whereas Class 3 mutants are rarely CMS1, and Class 2 mutants fall into either group. Relating *BRAF* mutation types to underlying mutational signatures, it is likely that Class 1 *BRAF* mutants are strongly selected (since V600E changes are not commonly created), and that Class 2 or 3 mutants are under weaker selective constraints in the distal colorectum, where they are over-represented.

Since codon 466 Class 3 mutations appear to be outliers (e.g. usually located proximally), we explored an ‘ABC’ classifier based on functional domains within BRAF (Supplementary Data). Similar strength associations to the ‘Class 1-2-3’ groups were found. It is possible that the classification of *BRAF* driver mutations will be refined over time and, we speculate, that a further group, principally comprising codon 466 changes, will emerge.

The combination of atypical *BRAF* and other atypical Ras mutations (in *KRAS, NRAS* or *NF1*) is consistent with a ‘mini-driver’ model in which some cancers acquire sufficient Ras activation through a two-step process rather than a single ‘hit’ at *BRAF* V600E or one of the common *KRAS* hotspots. In support of this suggestion, Class 2 and 3 changes appear to be favoured over Class 1 by the mutational processes active in the cancer cells. Our cohorts are insufficient to determine the order of acquisition of *BRAF* and additional Ras. We further explored whether atypical *KRAS* mutations presented with concomitant Ras/Raf at frequencies similar to that of atypical *BRAF*, but the former was significantly less frequent, perhaps reflecting the fact that atypical *KRAS* mutants possess some increased kinase activity in contrast to the loss of function associated with Class 3 *BRAF* mutations.

In an attempt to mitigate potentially confounding variables present in previous studies, we used mixed effects Cox proportional-hazards models accounting for covariables (e.g. age, sex, tumour stage, MSI subtype, location, and study) to analyse survival. Like previous work (5–7,36), we observe better overall survival for patients with a Class 3 *BRAF* mutation versus Class 1 (V600E). Our data suggest, however, that this benefit may be reduced, or absent, for cancers carrying both atypical *BRAF* and additional Ras mutations. Previously, Yaeger *et al*. (1) had performed a retrospective, multi-centre study to characterise the response of non-V600E *BRAF* variants to a range of anti-EGFR treatment regimens, querying datasets from five centres for metastatic CRC patients with non-V600E *BRAF* between 2010 and 2017. They found an 8% and 50% response rate for Class 2 and 3 mutations, respectively. A possible rationale for this difference in response rates is that inhibition of EGFR leads to a reduction in Ras signalling and thus inhibition of kinase-dead Class 3 *BRAF* mutants, which still require phosphorylation by Ras-GTP. Class 2 mutant *BRAF*, however, functions as Ras-independent homodimers and thus inhibition of upstream Ras would have little effect. As little under half of *BRAF* Class 3 CRCs in our analysis carry additional pathogenic Ras pathway mutations, we speculate that the efficacy of anti-EGFR therapy in this subset of double-mutant patients may be diminished.

Our work levers a large, combined dataset in conjunction with a comprehensive molecular evaluation of relevant Ras pathway genes, as opposed to focusing upon primarily hotspot mutations. The use of multiple datasets is almost unavoidable for studying rare driver mutation genotypes, and hence providing precision medicine for the patients concerned. We recognise, nonetheless, that our study cohorts may be heterogeneous in their methods, samples, inclusion criteria and data collected. There is certainly an argument for large clinicopathological-molecular studies of CRC patients with Class 2 or 3 *BRAF* mutations.

In summary, our findings shed further light on the classes of atypical *BRAF* mutations in colorectal cancer, which differ both molecularly and pathologically from typical *BRAF*-mutant disease. We believe these finding highlights the importance of whole-gene sequencing of patient samples in the clinic, with many atypical (and likely actionable) pathogenic mutations falling outside routinely assayed hotspot regions. With non-V600E *BRAF* mutant CRCs gaining interest due to the apparent positive prognosis, we hope that our work will stimulate clinical consideration of the potential confounding effects of additional, often non-canonical, Ras pathway mutations in this subset of disease.

## Supplementary Material

1

2

3

4

5

6

7

8

9

10

11

12

13

14

15

16

## Figures and Tables

**Table 1:** Clinicopathological and molecular summary information for *BRAF*-mutant patients in the combined cohort, stratified by functional Classes 1, 2, and 3. Presence of concomitant pathogenic mutations in *KRAS, NRAS*, or *NF1* is shown for each class. In addition to the data in the table, a single instance of co-occurring Class 2 and 3 *BRAF* was found. Unclassified mutations are assumed to be passenger changes for the purpose of our analysis. Wildtype *BRAF* CRCs within the combined cohort are included for reference. Percentages shown in parentheses reflect the proportion of total samples with available data for a given variable.

*BRAF* Mutation Class	Mutated Tumours	% of profiled tumours in dataset	% of *BRAF* mutant tumours in dataset	Tumours with pathogenic *KRAS,* *NRAS,* or *NF1* mutation	*KRAS*, *NRAS*, or *NF1* which are atypical	Tumours with no pathogenic *KRAS*, *NRAS*, or *NF1* mutation	Male	Female	Mean Age	MSI	MSS	Stage I	Stage II	Stage III	Stage IV	Proximal colon	Distal colon and rectum
**1**	709	10.7%	79.9%	17 (2.4%)	13 (82%**^+^**)	692	239 (36.5%)	416 (63.5%)	70.8	275 (52.4%)	250 (47.6%)	50 (10.1%)	192 (38.9%)	185 (37.5%)	67 (13.6%)	452 (83.7%)	88 (16.3%)
**2**	31	0.47%	3.49%	9 (29.03%)	5 (55.6%)	22	17 (63%)	10 (37%)	67.3	0 (0%)	23 (100%)	3 (18.8%)	6 (37.5%)	6 (37.5%)	1 (6.25%)	9 (37.5%)	15 (62.5%)
**3**	81	1.22%	9.13%	37 (45.7%)	29 (78%)	44	41 (58.6%)	29 (41.4%)	68.1	0 (0%)	69 (100%)	4 (8%)	9 (18%)	24 (48%)	13 (26%)	25 (43.9%)	32 (56.1%)
**Unclassified**	66	0.99%	7.44%	33 (50%)	14 (21.2%)	33	31 (64.6%)	17 (35.4%)	60.8	12 (27.9%)	31 (72.1%)	9 (20.5%)	17 (38.6%)	15 (34.1%)	3 (6.8%)	23 (50%)	23 (50%)
**Total *BRAF-*** ***mutant***	887	13.4%	100.0%	95 (10.7%)	62 (6.99%)	792	328 (41%)	472 (59%)	69.8	287 (43.5%)	373 (56.5%)	741 (16.5%)	1409 (31.4%)	1624 (36.2%)	707 (15.8%)	509 (76.3%)	158 (23.7%)
***BRAF-*** ***wildtype***	5718	86.6%	0%	3043 (53.2%)	585 (19.2%)	2675	2992 (58.1%)	2155 (41.9%)	66.2	1681 (39.5%)	2570 (60.5%)	675 (17.4%)	1185 (30.6%)	1394 (35.6%)	623 (16.1%)	1414 (34.3%)	2709 (65.7%)

+ All atypical Ras mutations within Class 1 were predicted pathogenic inactivation of *NF1*

**Table 2:** Survival analysis of BRAF-mutant CRC Classes: Multivariate and Univariate Mixed-effects Cox Proportional Hazards Models. Model I compares *BRAF* classes across all patients irrespective of additional Ras mutation; Model II compares Class 1 without Ras vs Class 3 with Ras mutations; Model III examines additional Ras mutation status within Class 3. All models include study as a random effect. Both univariate and multivariate analyses were performed on identical patient cohorts per model to ensure direct comparability. †: Variable not included in model specification. ¶: Variable could not be reliably estimated in Model III due to small subgroups.

	Model I				Model II				Model III			
Sample size	247				238				27			
Events	90				93				14			
Variable	Multivariate HR (95% CI)	P	Univariate HR (95% CI)	P	Multivariate HR (95% CI)	P	Univariate HR (95% CI)	P	Multivariate HR (95% CI)	P	Univariate HR (95% CI)	P
**BRAF Classification**												
Class 1	[Reference]	-	[Reference]	-	[Reference]	-	[Reference]	-	†	-	†	-
Class 2	1.13 (0.32 - 3.94)	0.85	1.36 (0.42 - 4.41)	0.61	-	-	-	-	†	-	†	-
Class 3	0.25 (0.08 - 0.73)	0.01	0.4 (0.13 - 1.21)	0.11	0.94 (0.46 - 1.91)	0.86	1.09 (0.54 - 2.19)	0.8	†	-	†	-
**Clinical Features**												
Age at Diagnosis	1.01 (0.98 - 1.03)	0.68	0.99 (0.96 - 1.01)	0.22	1.00 (0.98 - 1.02)	0.99	0.98 (0.96 - 1)	0.12	1.04 (0.93 - 1.16)	0.52	0.97 (0.91 - 1.03)	0.29
Sex (Female)	[Reference]	-	[Reference]	-	[Reference]	-	[Reference]	-	[Reference]	-	[Reference]	-
Sex (Male)	1.30 (0.82 - 2.07)	0.27	1.15 (0.73 - 1.80)	0.56	1.24 (0.79 - 1.97)	0.35	1.16 (0.75 - 1.81)	0.5	1.67 (0.20 - 14.1)	0.48	2.61 (0.68 - 10.1)	0.16
Left-sided	[Reference]	-	[Reference]	-	[Reference]	-	[Reference]	-	[Reference]	-	[Reference]	-
Right-sided	0.97 (0.51 - 1.87)	0.93	1.39 (0.75 - 2.57)	0.3	1.06 (0.56 - 2.03)	0.85	1.28 (0.68 - 2.40)	0.44	0.60 (0.14 - 2.50)	0.64	0.73 (0.23 - 2.31)	0.59
**Disease Staging**												
Stage I	[Reference]	-	[Reference]	-	[Reference]	-	[Reference]	-	[Reference]	-	[Reference]	-
Stage II	1.32 (0.29 - 5.93)	0.72	1.32 (0.30 - 5.82)	0.72	2.44 (0.32 - 18.96)	0.39	2.41 (0.31 - 18.6)	0.4	¶	-	¶	-
Stage III	2.91 (0.69 - 12.38)	0.15	2.47 (0.59 - 10.4)	0.22	5.21 (0.71 - 38.3)	0.11	5.41 (0.74 - 39.7)	0.1	¶	-	¶	-
Stage IV	16.6 (3.78 - 73.2)	<0.001	14.2 (3.31 - 61)	<0.001	28 (3.77 - 207.5)	<0.001	35.2 (4.8 - 256)	<0.001	¶	-	¶	-
**Additional Features**												
MSI	[Reference]	-	[Reference]	-	[Reference]	-	[Reference]	-	[Reference]	-	[Reference]	-
MSS	1.39 (0.80 - 2.42)	0.25	1.12 (0.63 - 2)	0.69	1.41 (0.81 - 2.44)	0.23	1.46 (0.81 - 2.62)	0.21	¶	-	¶	-
**Additional Ras Status**												
No Additional Ras	†	-	†	-	†	-	†	-	[Reference]	-	[Reference]	-
Additional Ras	†	-	†	-	†	-	†	-	4.92 (1.12 - 21.6)	0.03	4.17 (1.16 - 14.9)	0.03

## Data Availability

Data obtained from public cohorts are available through the *cBioPortal for Cancer Genomics* (https://www.cbioportal.org) using the study codes referenced in the methods section. In order to preserve patient privacy across the non-public cohorts and comply with reporting restrictions, aggregate clinicopathological and mutational data tables are presented in this manuscript. Data accessibility for non-public cohorts is as such: non-public data generated through use of *Genomics England 100,000 Genomes Project* samples are available following registration within the Genomics England research environment (https://www.genomicsengland.co.uk/research/research-environment) and request to the corresponding author. The non-public data in this publication generated by the S:CORT Consortium are available for use by not-for-profit organisations for academic, teaching, and educational purposes on request. The S:CORT data are available for commercial use, on commercial terms, via Cancer Research Horizons https://www.cancerresearchhorizons.com/. Data from the remaining non-publicly available cohorts, IMAGINE (ISRCTN42303887) and QUASAR 2 (ISRCTN45133151), can be requested from the steering committee or chief investigator of the clinical trial concerned.

## Implications

The heterogeneous nature of *BRAF*-mutant CRCs, particularly among Class 2/3 mutations which frequently harbour additional Ras mutations, highlights the necessity of comprehensive molecular profiling.
