# Coevolution of Atypical *BRAF* and *KRAS* Mutations in Colorectal Tumorigenesis

**DOI:** 10.1158/1541-7786.MCR-24-0464

**Published:** 2025-01-03

**Authors:** Connor E. Woolley, Enric Domingo, Juan Fernandez-Tajes, Kathryn A.F. Pennel, Patricia Roxburgh, Joanne Edwards, Susan D. Richman, Tim S. Maughan, David J. Kerr, Ignacio Soriano, Ian P.M. Tomlinson

**Affiliations:** 1 Department of Oncology, University of Oxford, Oxford, United Kingdom.; 2 School of Cancer Science, Wolfson Wohl Cancer Research Centre, University of Glasgow, Glasgow, United Kingdom.; 3 Division of Pathology and Data Analytics, University of Leeds, Leeds, United Kingdom.; 4 Radcliffe Department of Medicine, University of Oxford, Oxford, United Kingdom.

## Abstract

*BRAF* mutations in colorectal cancer comprise three functional classes: class 1 (V600E) with strong constitutive activation, class 2 with pathogenic kinase activity lower than that of class 1, and class 3 which paradoxically lacks kinase activity. Non–class 1 mutations associate with better prognosis, microsatellite stability, distal tumor location, and better anti-EGFR response. An analysis of 13 colorectal cancer cohorts (*n* = 6,605 tumors) compared class 1 (*n* = 709, 10.7% of colorectal cancers), class 2 (*n* = 31, 0.47%), and class 3 (*n* = 81, 1.22%) mutations. Class 2–mutant and class 3–mutant colorectal cancers frequently co-occurred with additional Ras pathway mutations (29.0% and 45.7%, respectively, vs. 2.40% in class 1; *P* < 0.001), often at atypical sites (*KRAS* noncodon 12/13/61, *NRAS*, or *NF1*). Ras pathway activation was highest in class 1 and lowest in class 3, with a greater distal expression of EGFR ligands (amphiregulin/epiregulin) supporting weaker *BRAF* driver mutations. Unlike class 1 mutants, class 3 tumors resembled chromosomally unstable colorectal cancers in mutation burdens, signatures, driver mutations, and transcriptional subtypes, whereas class 2 mutants displayed intermediate characteristics. Atypical *BRAF* mutations were associated with longer overall survival than class 1 mutations (HR = 0.25; *P* = 0.011) but lost this advantage in cancers with additional Ras mutations (HR = 0.94; *P* = 0.86). This study supports the suggestion that class 3 *BRAF* mutations amplify existing Ras signaling in a two-mutation model and that the enhancement of weak/atypical Ras mutations may suffice for tumorigenesis, with potentially clinically important heterogeneity in the class 2/3 subgroup.

**Implications:**

The heterogeneous nature of *BRAF*-mutant colorectal cancers, particularly among class 2/3 mutations which frequently harbor additional Ras mutations, highlights the necessity of comprehensive molecular profiling.

## Introduction

**Figure 1.**
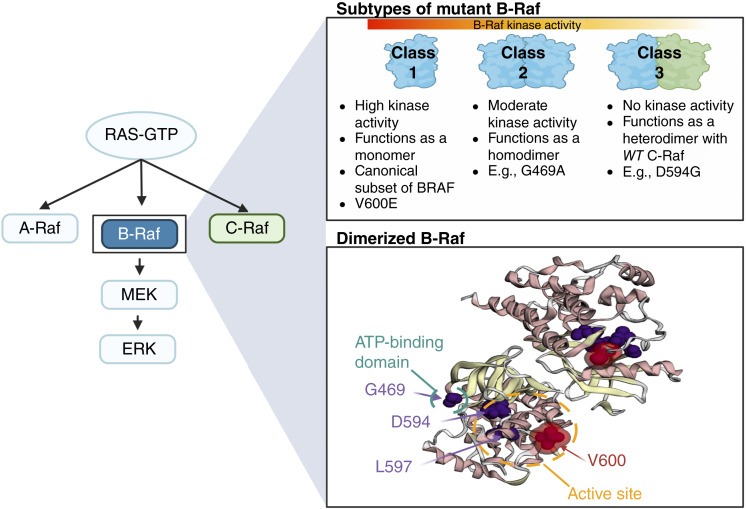
*BRAF* mutations are classified by their effects on kinase activity. B-Raf is a central component of the Ras signaling cascade, and mutations in *BRAF* are termed either class 1, 2, or 3 based on the resultant kinase activity. Mutations in *BRAF* are most commonly localized to the ATP-binding domain and active site. (Created with BioRender.com.)

**Figure 2.**
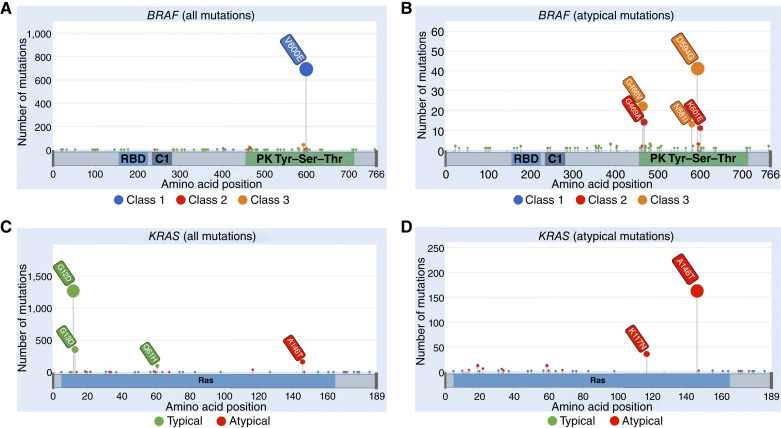
Lollipop plots representing *BRAF* and *KRAS* mutations across the combined cohorts. **A,***BRAF* mutations showing the predominance of V600E. **B,***BRAF* mutation distribution with absent V600E shows that the second most frequent alterations are class 3 changes (e.g., D594G and G466V). **C,***KRAS* mutations showing the predominance of “typical” changes at codons 12 and 13. **D,** The distribution of *KRAS* mutations classified as “atypical” in our analysis. Plots were generated using the g3viz R package. Note that these mutation frequencies do not necessarily correspond precisely to those in unselected colorectal cancers owing to the inclusion criteria for several studies, especially those based on clinical trials. RBD, Ras-binding domain; Ser, serine; Thr, threonine; Tyr, tyrosine. (Created with BioRender.com.)

Many colorectal cancers acquire driver mutations in genes in the MAPK/ERK signaling pathway, including heterozygous missense changes in *KRAS*, *NRAS*, or *BRAF*. The most common *BRAF* driver mutation in colorectal cancer is a valine to glutamic acid substitution, V600E, that causes constitutive Ras pathway activation downstream of BRAF (Fig. 1). V600E acts as a monomer and is termed a class 1 mutation (1). There also exist class 2 *BRAF* mutations (e.g., at codons 469, 597, and 601) that produce active BRAF homodimers. However, an important minority of colorectal cancers harbor class 3 mutations in *BRAF* that paradoxically lead to a kinase-impaired protein (2), the most common being amino acid substitutions at codons 466, 594, and 596. Class 3 mutations seem to act by increasing pathway activation through enhanced stabilization of BRAF–CRAF heterodimers in the presence of active Ras (2–4). Whereas class 1 mutations tend to occur in colorectal cancers of the proximal colorectum, class 2 and 3 mutations may be overrepresented in distal colorectal cancers and might confer a relatively good prognosis (5–7). Further details of the locations of mutations of each *BRAF* class are provided in Materials and Methods and Fig. 2.

The spectrum of driver mutations found in any tumor depends on both the Darwinian selection (dependent on the cell’s genetic complement and tumor microenvironment) and the mutational processes active within the cell. The latter manifest in the overall spectrum of mutations in the tumor, which can be identified through large-scale DNA sequencing. As multiple mutational processes may act and vary in time and space, the mutation spectrum can be deconvolved into signatures, most often based on the 96 types of base substitutions in a trinucleotide (3nt) context (9). Individual signatures may result from exposure to carcinogens, specific defects in DNA repair, or other cellular features or processes. As a result, it is possible to infer the likelihood of a particular driver mutation arising in a cancer by comparing its 3nt context with the mutation spectrum and dominant mutational signatures present in that cancer. In the case of colorectal cancer, our previous work has shown that *BRAF*^V600E^ (c.1799T>A) mutations are mutationally unlikely to occur, because they arise from a mutational channel (GTG>GAG) with low activity (10). Their high prevalence suggests that they are strongly positively selected, presumably because they efficiently activate the MAPK pathway.

The existence of class 2 and 3 *BRAF* driver mutations, with reduced or absent kinase activity, is therefore an apparent paradox. We can envisage at least three explanations that are not mutually exclusive: (i) mutational processes strongly favor the occurrence of class 2/3 versus class 1 mutations in a minority of cancers; (ii) the differential selective advantage between class 2/3 and class 1 mutations is low in some microenvironments; and (iii) the relative weakness of class 2/3 mutations is compensated by other factors, such as extra driver mutations in other genes. In this study, we use 13 colorectal cancer cohorts (Supplementary Fig. S1) with genome, exome, or panel sequencing data to investigate the mutational landscape of colorectal cancers with class 2 or 3 *BRAF* mutations.

## Materials and Methods

### Data acquisition

Data on Ras pathway mutations in colorectal cancer were obtained from the following studies or repositories (details in Supplementary Table S1): Stratification in Colorectal Cancer (S:CORT) Consortium (11), The Cancer Genome Atlas (TCGA; RRID: SCR_003193; ref. 12), the Genomics England 100,000 Genomes Project (RRID: SCR_010502; ref. 13), QUASAR 2 (14), IMAGINE (15), and the cBioPortal for cancer genomics (16) which was used to access publicly available cohorts such as “coadread_dfci_2016,” “coadread_cptac_2019,” “coadread_casecc_2015,” “coadread Genentech,” “coadread_mskcc,” “coadread_tcga_pan_cancer,” “crc_msk_2017,” “rectal_msk_2019,” and “crc_apc_impact_2020” (12, 17–23). Any duplicate sample identifiers across studies were removed. All data sets had identified somatic mutations in the most frequent Ras pathway drivers in cancer, *BRAF*, *KRAS*, *NRAS*, and *NF1*, by direct sequencing of all relevant exons, and many provided microsatellite instability (MSI) status and routine clinicopathologic data, including cancer location in the colorectum. Additional data, specifically mutations in other major and minor colorectal cancer driver genes and somatic copy-number changes, were available from some data sets (Supplementary Fig. S1; Supplementary Table S1).

### Analysis of the driver status of non-*BRAF*^V600E^ mutations using dNdScv

To investigate whether *BRAF* mutations, excluding the common V600E variant, occur under positive selection, we performed dN/dS analysis using dNdScv (RRID: SCR_001905; ref. 24) as part of the IntOGen driver analysis Nextflow pipeline within the 100KGP Genomics England Research Environment. Following the methods described by Cornish and colleagues (25), we created separate datasets for microsatellite stable (MSS) and MSI colorectal cancers, with V600E variants removed from both groups. This approach allowed us to specifically examine the selection pressure on non-*BRAF*^V600E^ mutations, of which the majority in the residual cohort were missense mutations leading to class 2 or class 3 *BRAF*.

### Class 1, 2, and 3 *BRAF* driver mutations

Based on their overrepresentation above background frequencies in multiple cancer types and specific *in vitro* functional assessments in the literature (2), we refer to the following codons when classifying *BRAF* mutations:

Class 1: 600
Class 2: 464, 469, 597, and 601
Class 3: 466, 581, 594, 595, and 596

Class 2 and 3 *BRAF* mutations are collectively denoted as “atypical” *BRAF* mutations below. Mutations occurring in codons other than the above were excluded from class-based analyses, as data are lacking on their functional dependence on Ras signaling and dimerization. In exploratory analyses, we also examined *BRAF* mutations according to their locations in functional domains of the *BRAF* protein, as shown in Fig. 2: A, codon 600; B, flanking codon 600 (581–601, excluding 600); and C, within the ATP-binding site (464, 466, and 469).

### Classification of “typical” and “atypical” driver mutations in *KRAS*, *NRAS*, and *NF1*

Driver mutations in Ras pathway genes other than *BRAF* were assessed. These mutations most commonly occurred in the genes *KRAS*, *NRAS*, and *NF1*. Therefore, we focused upon these genes rather than other, rarely mutated genes, such as the ErbB ligands, which have the additional issue that their mutation pathogenicity is often uncertain. Mutated codons in *KRAS*, *NRAS*, and *NF1* (collectively referred to as “other Ras pathway driver genes” herein) were prespecified as “typical” or “atypical” through the interrogation of the Catalog of Somatic Mutations in Cancer database (26) based on their overrepresentation above background frequencies in multiple cancer types, specific functional assessments, and frequencies in colorectal cancer. Synonymous or probably nonpathogenic mutations were not considered. For the purposes of this study, we classified *typical* pathogenic *KRAS* mutations as amino acid substitutions occurring at codons 12, 13, and 61, whereas *atypical* (yet predicted pathogenic) *KRAS* mutations were at codons 14, 19, 22, 33, 34, 59, 60, 68, 117, 146, and 147. Atypical *KRAS* mutations are often weaker drivers of Ras signaling (e.g., L19F and A146T) than typical drivers (e.g., G12D); however, these mutations still result in pathogenic Ras pathway stimulation (8, 27). All predicted pathogenic *NRAS* mutations (codons 12, 13, and 61) and *NF1* (protein-truncating) mutations were also classed as *atypical* Ras pathway mutations based on the relatively low frequency of any pathogenic mutations in these genes in colorectal cancer (28).

### Copy-number analysis and clonality estimates

Statistical analysis of copy-number alterations (CNA) was conducted through the *enrichments* package included in the *cBio Cancer Genomics Portal* software package for the S:CORT and cBioPortal datasets. For 100KGP, the *Battenberg* program (RRID: SCR_017098; ref. 29) was used to call the allele-specific copy number from whole-genome sequencing (WGS) data based on cancer cell purity estimates from CCube (bioRxiv 484402). As CNA calls from Battenberg are based on germline polymorphisms, they do not attribute a copy number to the mutant or wild-type (WT) allele at oncogenes such as *BRAF*, *KRAS*, or *NRAS*. An expression (Eq. A) was therefore derived to assign the copy number of each oncogene allele. If *VAFMut* is the frequency of the mutant allele in the cancer sequencing reads, *Nmut* is the putative copy number of the mutant allele, *Nwtcan* is the copy number of the WT allele, two represents the number of copies of the WT allele in a normal cell, and purity represents the calculated tumor purity (proportion of cancer cells in the sample). The substitution of each allele-specific copy-number value into Eq. A as either *Nmut* or *Nwtcan* in turn generally provides only one valid solution to the CCube-estimated tumor purity, thus allowing the assignment of *Nmut* and *Nwtcan*.

$$Purity=\frac{2}{\left(\frac{Nmut}{VAFMut}\right)-Nmut-Nwtcan+2}$$ (A)

### Assessing mutation clonality in tumors

For patients whose archival formalin-fixed, paraffin-embedded tissues were available, three 10-μm sections were microdissected with a needle into multiple regions for extraction and processed for DNA using Roche High Pure FFPET DNA Kit as per the manufacturer’s recommended protocol. Purified DNA was eluted into 50 μL, and target exons for *BRAF* and concomitant Ras (*KRAS*/*NRAS*) were amplified with high-fidelity polymerase (Q5 Hot Start HiFi Polymerase, New England Biolabs). Primers for amplification can be found in Supplementary Table S2. Annealing temperatures were determined via the New England Biolabs Tm Calculator (https://tmcalculator.neb.com/), and an extension time of 30 seconds was used. PCR amplicons were purified using Qiagen QIAquick PCR Purification Kit and eluted into 30 μL prior to submission for Sanger sequencing using the same forward and reverse primer pairs for initial amplification. Sequencing chromatograms were visualized in SnapGene.

### Mutation spectra and signatures

The *SigProfilerExtractor* package (RRID: SCR_023121; ref. 30) was used to produce genome-wide 96-channel mutation spectra for WGS samples in the 100KGP dataset. Mutations are presented in a 3nt context, in which each 3nt context represents a mutational channel, which is reported from the pyrimidine strand for conciseness. With raw 96-channel counts called for each sample, they were normalized to proportional activity within the sample and thus summed to one, allowing comparison between samples. The mutation spectrum for each sample was fitted against Catalog of Somatic Mutations in Cancer Single Base Substitution signatures (version 3; ref. 31) to deconvolve the contributing signatures and thus infer the mutational processes undergone by the tumor throughout its lifetime.

### Logistic regression analysis of mutation channel activities

Logistic regression was performed in R (version 4.2.1; RRID: SCR_001905) using the base *glm* package, with models incorporating the explanatory variables “casual channel proportion” or “V600E channel proportion.” Causal/V600E channel proportions were calculated as the proportion of 3nt channel mutations of all 3nt channel mutations within the sample responsible for generating the *BRAF* mutation. Covariates included in all models were tumor location (as either proximal or distal colorectum), MSI status, age at diagnosis, and sex.

### Regional expression analysis of Ras/EGFR pathway genes in normal colorectal tissues

Differential gene expression analysis across core Ras/Raf pathway genes *KRAS*, *BRAF*, and *NRAS*, plus the EGFR ligand genes amphiregulin (*AREG*) and epiregulin (*EREG*), was conducted across normal RNA sequencing (RNA-seq) samples from Fernandez-Rozadilla and colleagues (32) and subsequently validated using normal samples present in TCGA COADREAD cohorts (12). Expression analysis was conducted in R (4.2.1) using DESeq2 (1.4.2; RRID: SCR_015687; ref. 33) with tissue location as the condition variable. Only samples with specific anatomic location available were included, with distal colon and rectum merged to create a distal/rectal grouping for contrast against the proximal colon. Multiple testing corrections were performed using the Benjamini–Hochberg procedure.

### Gene set enrichment analysis

The gene set enrichment analysis (GSEA) tool (version 4.1.0; RRID: SCR_003199) was obtained from the Broad Institute’s GSEA portal (http://www.gsea-msigdb.org/gsea/index.jsp), and gene signatures were extracted from MSigDb (http://www.gsea-msigdb.org/gsea/msigdb/index.jsp; RRID: SCR_016863). Analysis made use of the curated oncogenic pathways (C6) with 10,000 permutations. All other parameters were set to their default values, with results considered significant at an FDR-adjusted *q* value of 0.05 and a normalized enrichment score of < −1.2 or >1.2, depending upon signature directionality.

### Consensus molecular subtypes by *BRAF* class

**Figure 3.**
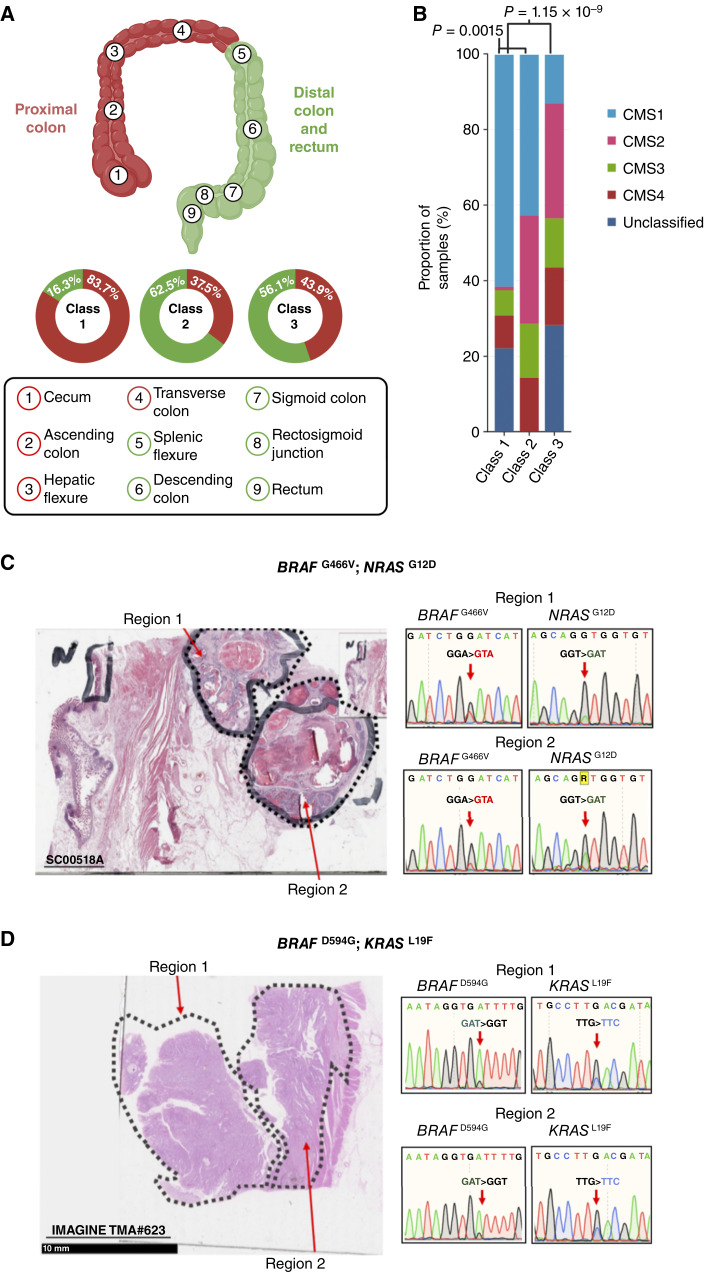
Classes of *BRAF* mutation exhibit distinct clinicopathologic and molecular characteristics. **A,** Subdivision of the large bowel into the proximal and distal colorectum. Proportions of colorectal cancers in each location are shown by *BRAF* mutation class. **B,** CMS classifications of *BRAF*-mutant colorectal cancers by mutation class. The differences between class 2 or 3 versus class 1 (V600E) were highly significant (*P* < 0.001). No significant difference was observed between classes 2 and 3 (*P* = 0.270). **C** and **D,** Sanger sequencing chromatograms highlighting the spatial co-occurrence of *BRAF* and additional Ras pathway mutations in two colorectal cancers. Two regions of each cancer were microdissected, DNA was extracted, and relevant exons were PCR-amplified prior to Sanger sequencing. Illustrative results are shown. The co-occurrence of *BRAF*^G466V^ and *NRAS*^G12D^ can be observed in tumor 1 (**C**), and of *BRAF*^D594G^ and *KRAS*^L19F^ in tumor 2 (**D**). This analysis does not exclude fine-scale spatial mixing of distinct subclones but is consistent with the two mutations tested being present in the same tumor cells. (Created with BioRender.com.)

Consensus molecular subtypes (CMS) were called as part of the S:CORT Consortium workflow using transcriptomic array data generated via the Almac XCel array. Initial comparisons between classes as shown in Fig. 3B were performed by Fisher exact tests. Multiple logistic regression models controlling for covariates were produced in R using the base *glm* package, with each CMS group used as the outcome variable. *BRAF* class and CMS groups were treated as categorical variables and dummy coded, with each CMS group modeled per *BRAF* class. Covariates included in all models were age at diagnosis, tumor location (proximal or distal colorectum), MSI status, and stage.

### Survival analysis

Statistical analyses based on survival data were performed in R (version 4.2.1; ref. 34) using mixed-effects Cox proportional hazards models through the *coxme*, *survminer* (RRID: SCR_021904), and *survival* packages. Cohort studies used for survival analysis with overall survival (OS) data available were the 100,000 Genomes Project colorectal cancer, S:CORT, TCGA COADREAD, CPTAC 2019, colorectal cancer MSK 2017, and colorectal cancer APC IMPACT 2020. For all mixed-effects Cox models, the following were included as fixed-effects covariates: age at diagnosis, sex, tumor location (left/right), and cancer stage. The study cohort was included in all models as a random-effect covariate. Proportional hazards assumptions were tested using scaled Schoenfeld residuals from corresponding fixed-effects models. In model I (survival by *BRAF* class irrespective of additional Ras), stage showed a minor violation (*P* = 0.017) driven by sparse events at late follow-up (Supplementary Fig. S2A). Given the clinical importance and stable effect of stage during the majority of follow-up, we retained it in the final models, interpreting its effect as an average over time. No violation of proportional hazards was observed for stage in model II (*P* = 0.109; Supplementary Fig. S2B), whereas model III (the effect of additional Ras status within class 3 *BRAF*-mutant colorectal cancers) showed a stronger stage violation (*P* = 0.001, Supplementary Fig. S2C) because of the limited sample size in the class 3–only cohort, precluding the interpretation of stage effects. The main variable of interest (additional Ras status) met proportional hazards assumptions (*P* = 0.18), allowing valid interpretation of these results.

### Data availability

Data obtained from public cohorts are available through the *cBioPortal for Cancer Genomics* (https://www.cbioportal.org) using the study codes referenced in the methods section. In order to preserve patient privacy across the nonpublic cohorts and comply with reporting restrictions, aggregate clinicopathologic and mutational data tables are presented in this article. Data accessibility for nonpublic cohorts is as such: nonpublic data generated through the use of *Genomics England 100*,*000 Genomes Project* samples are available following registration within the Genomics England research environment (https://www.genomicsengland.co.uk/research/research-environment) and request to the corresponding author. The nonpublic data in this publication generated by the S:CORT Consortium are available for use by not-for-profit organizations for academic, teaching, and educational purposes on request. The S:CORT data are available for commercial use on commercial terms via Cancer Research Horizons https://www.cancerresearchhorizons.com/. The data from the remaining nonpublicly available cohorts, IMAGINE (ISRCTN42303887) and QUASAR 2 (ISRCTN45133151), can be requested from the steering committee or chief investigator of the clinical trial concerned.

## Results

### Class 2 and 3 *BRAF* mutations show positive selection in colorectal cancer and frequently co-occur with other Ras pathway mutations

To explore the driver status of non-*BRAF*^V600E^ mutations in colorectal cancer, particularly classes 2 and 3, we used dNdScv analysis (24), which identifies genes under significant selection pressure within cancer cohorts. A dN/dS ratio exceeding one indicates positive selection of nonsynonymous mutations, suggesting potential driver status. Using the 100KGP WGS cohort, we analyzed MSS and MSI primary colorectal cancers separately (as described by Cornish and colleagues; ref. 25) after excluding V600E variants. The remaining non-*BRAF*^V600E^ variants predominantly comprised missense substitutions resulting in class 2 or class 3 mutations. In MSS primary tumors, non-*BRAF*^V600E^ missense mutations showed significant positive selection (dN/dS = 5.43; *q* = 0.012). In contrast, MSI tumors showed no significant positive selection for non-*BRAF*^V600E^ mutations (missense dN/dS = 1.14; *q* = 0.99). Separate analyses of class 2 and 3 mutations independently showed nominally significant positive selection of *BRAF* in MSS tumors for both classes (dN/dS = 7.21; *P* = 0.013 and dN/dS = 8.51; *P* = 0.005, respectively).

The *KRAS* and *BRAF* mutations found in the combined cohorts are shown in Fig. 2. Compared with tumors harboring class 1 mutations, colorectal cancers with atypical *BRAF* mutations in classes 2 and 3 tended to occur in the distal large bowel (Table 1; Fig. 3A), with 58% of tumors occuring in the distal colon versus 16.3% in the proximal colorectum (*P *< 0.001, Fisher exact test), consistent with previous reports (5, 7, 35). There was no significant difference in the location between classes 2 and 3 (62.5% vs. 56.1% in distal colorectum; *P* = 1.0, Fisher exact test). Further exploration of the class 3 cohort alone showed that mutations at codon 466 were strongly associated with proximal location, in contrast to other class 3 variants (13/18 vs. 12/39, respectively; *P* = 0.005, Fisher exact test). No significant differences were observed between the age of sampling and *BRAF* mutation class.

**Table 1.** Clinicopathologic and molecular summary information for *BRAF*-mutant patients in the combined cohort, stratified by functional classes 1, 2, and 3. The presence of concomitant pathogenic mutations in *KRAS*, *NRAS*, or *NF1* is shown for each class. In addition to the data in the table, a single instance of co-occurring class 2 and 3 *BRAF* mutations was found. Unclassified mutations are assumed to be passenger changes for the purpose of our analysis. WT *BRAF* colorectal cancers within the combined cohort are included for reference. Percentages shown in parentheses reflect the proportion of total samples with available data for a given variable.

*BRAF* mutation class	Mutated tumors	% of profiled tumors in dataset	% of *BRAF*-mutant tumors in dataset	Tumors with pathogenic *KRAS*, *NRAS*, or *NF1* mutation	*KRAS*, *NRAS*, or *NF1* which are atypical	Tumors with no pathogenic *KRAS*, *NRAS*, or *NF1* mutation	Male	Female	Mean age	MSI	MSS	Stage I	Stage II	Stage III	Stage IV	Proximal colon	Distal colon and rectum
1	709	10.7%	79.9%	17 (2.4%)	13 (82%^a^)	692	239 (36.5%)	416 (63.5%)	70.8	275 (52.4%)	250 (47.6%)	50 (10.1%)	192 (38.9%)	185 (37.5%)	67 (13.6%)	452 (83.7%)	88 (16.3%)
2	31	0.47%	3.49%	9 (29.03%)	5 (55.6%)	22	17 (63%)	10 (37%)	67.3	0 (0%)	23 (100%)	3 (18.8%)	6 (37.5%)	6 (37.5%)	1 (6.25%)	9 (37.5%)	15 (62.5%)
3	81	1.22%	9.13%	37 (45.7%)	29 (78%)	44	41 (58.6%)	29 (41.4%)	68.1	0 (0%)	69 (100%)	4 (8%)	9 (18%)	24 (48%)	13 (26%)	25 (43.9%)	32 (56.1%)
Unclassified	66	0.99%	7.44%	33 (50%)	14 (21.2%)	33	31 (64.6%)	17 (35.4%)	60.8	12 (27.9%)	31 (72.1%)	9 (20.5%)	17 (38.6%)	15 (34.1%)	3 (6.8%)	23 (50%)	23 (50%)
Total *BRAF-*mutant mutations	887	13.4%	100.0%	95 (10.7%)	62 (6.99%)	792	328 (41%)	472 (59%)	69.8	287 (43.5%)	373 (56.5%)	741 (16.5%)	1,409 (31.4%)	1,624 (36.2%)	707 (15.8%)	509 (76.3%)	158 (23.7%)
*BRAF-*WT	5,718	86.6%	0%	3,043 (53.2%)	585 (19.2%)	2,675	2,992 (58.1%)	2,155 (41.9%)	66.2	1,681 (39.5%)	2,570 (60.5%)	675 (17.4%)	1,185 (30.6%)	1,394 (35.6%)	623 (16.1%)	1,414 (34.3%)	2,709 (65.7%)

a All atypical Ras mutations within class 1 were predicted pathogenic inactivation of *NF1*.

The overall frequency of class 1 *BRAF* mutations, all bar one of which were V600E, in our combined data set was 10.7% (709/6605). Values per cohort are presented in Supplementary Table S3A. Largely as expected, V600E alterations were almost entirely mutually exclusive with other Ras pathway driver mutations (36–38). Seventeen colorectal cancers harboring a V600E mutation also presented with additional, predicted pathogenic mutations in *KRAS*, *NRAS*, or *NF1* (17/709, 2.40%), comprising 13 colorectal cancers with *NF1*-inactivating mutations and four with *KRAS* mutations (4/709, 0.71%, mostly G12D).

We then assessed the 81 (81/6605, 1.22%) colorectal cancers with a class 3 *BRAF* mutation (codons 466, 581, 594, 595, and 596). Thirty-seven (37/81, 45.7%) of these tumors additionally carried a pathogenic mutation in *KRAS*, *NRAS*, and/or *NF1* (Table 1). One class 3–mutant tumor had an additional *BRAF* mutation, harboring both D594G and a class 2 change, G469V. Of the 31 (31/6605, 0.47%) colorectal cancers with class 2 *BRAF* mutations (codons 464, 469, 597, and 601), nine had additional pathogenic Ras pathway mutations (9/31, 29.0%). The overrepresentation of additional Ras pathway driver mutations in colorectal cancers with class 3 *BRAF* mutations was very strong relative to class 1 *BRAF*-mutant colorectal cancers (45.7% vs. 2.40%; *P* < 0.001, Fisher exact test), and a slightly weaker association was present in class 2–mutant tumors relative to class 1 (29.0% vs. 2.40%; *P* < 0.001, Fisher exact test).

Focusing on the 37 colorectal cancers with class 3 *BRAF* mutants and concomitant Ras pathway mutations, only eight harbored typical *KRAS* mutations (*P* < 10^−5^, Fisher exact test). Notable atypical Ras pathway mutations in the 29 other tumors included 18 changes in *KRAS* (including V14I, L19F, P34L, G60D, K117Q, and A146V) and 12 in *NRAS*. One cancer had a class 3 mutation (G466V), two atypical *KRAS* mutations (V14L and D33E), and an *NRAS* mutation (G12V). Six of the nine additional Ras mutations in class 2 tumors were also atypical, comprising four *KRAS* changes (A59T, A146V, and A146T × 2) and two *NRAS* mutations (G12D and G12V). The frequency of atypical Ras pathway mutations in particular was higher than expected overall for class 2 and 3 *BRAF-*mutant cancers when compared with class 1 cancers (13/709 30.6% vs. 34/112 1.8%, respectively; *P* < 0.001, Fisher exact test; Table 1). There was also a borderline significant difference observed between class 2 and class 3 *BRAF*-mutant colorectal cancers (frequencies 16.1% and 35.8%, respectively; *P* = 0.06, Fisher exact test; Table 1).

**Figure 4.**
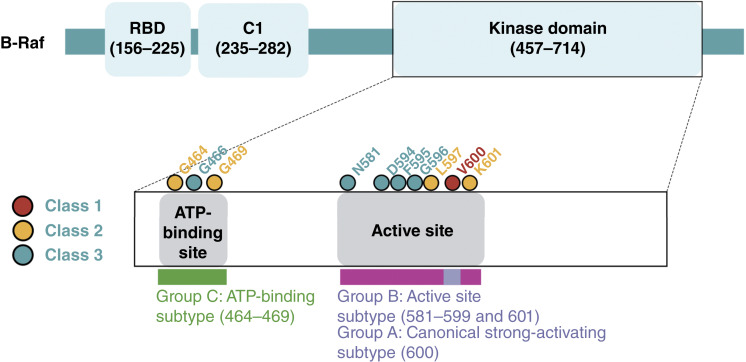
*BRAF* functional domains and comparison between the 1-2-3 and A-B-C *BRAF* classification systems. The 1-2-3 *BRAF* mutation classification is based on *in vitro* assays of Ras pathway activity. However, as it has not been validated by the analysis of native mutations in isogenic cells, we explored, in parallel, a simpler classification based on the mutation location in functional domains: A, codon 600; B, other active site mutations flanking codon 600 (581–601); and C, ATP-binding site mutations (464–469). RBD, Ras-binding domain. (Created with BioRender.com.)

The 1-2-3 *BRAF* mutation classification is based on *in vitro* assays of Ras pathway activity, although these were not native mutations in isogenic cells. We therefore considered, in parallel, a simpler classification based on the mutation location in functional domains: A, codon 600; B, flanking codon 600 (581–601, excluding 600); and C, within the ATP-binding site (464, 466, and 469; Fig. 4; Supplementary Tables S3B and S4). In agreement with the conventional classification, we found strongly significant associations between classes B and C and the presence of additional Ras pathway mutations in comparison with class A which mostly comprises V600E (2.40% for class A vs. 35.6% and 52.5% for B and C, respectively; *P* < 0.001, Fisher exact tests; Supplementary Table S4). Additional analyses using the exploratory A-B-C classification are presented in Supplementary Text S1.

### Clonality of atypical BRAF and additional Ras mutations

In Genomics England 100,000 Genomes Project colorectal cancers with either a class 2 or 3 *BRAF* variant and an additional Ras pathway variant, we investigated whether these mutations were present in different cancer subclones or in the same cell. By integrating the estimated purity of each tumor, the observed allele frequency of each mutation of interest, and the allele-specific copy numbers, we estimated a corrected allele frequency for the *BRAF* variant and the other Ras variant (details in Copy-number analysis and clonality estimates). We then used these estimates to correct mutant and reference read counts to the underlying copy number and tested whether the counts of the two variants differed significantly from the estimated tumor purity at nominal *P <* 0.05, thus suggesting whether they are clonal or subclonal and whether they are nested or entirely distinct. In 7/8 cancers analyzed, the data were consistent with atypical *BRAF* and concomitant Ras pathway mutations being present in the same clonal population (*χ*^2^ tests of independence, Supplementary Table S5).

We further obtained archival tumor material from two *BRAF*–Ras double-mutant colorectal cancers (Fig. 3C and D). Hematoxylin and eosin sections were dissected into multiple, spatially distinct regions within each tumor and directly Sanger sequenced for the relevant region of *BRAF* and either *KRAS* or *NRAS*, as appropriate. Sanger sequencing chromatograms sampled in Fig. 3 are provided in an accompanying zip archive as Supplementary Data S1. In both cases, there was concordance between the two mutations. We thus concluded that, in general, atypical *BRAF* mutations and concomitant additional Ras pathway mutations are likely to be present in the same cancer cells.

We also explored whether gene amplification was used by tumors to boost the putative weak effects of atypical *BRAF* mutations as an alternative to acquiring atypical Ras mutations. For samples harboring class 2 or 3 *BRAF* mutations, we identified no association between the absence of an additional Ras pathway mutation and the presence of a *BRAF* amplification (copy number >2; *P* = 1, Fisher exact test). We further found no significant differences in gene amplification when stratifying class 2 and class 3 *BRAF* mutants by the presence or absence of additional pathogenic mutation in *KRAS*, *NRAS*, or *NF1* (*P* > 0.05). These data suggest that even class 2 atypical *BRAF* mutations do not have strong enough intrinsic Ras activity to be boosted to pathogenic levels by copy number increase.

### Atypical *BRAF* mutations and additional Ras pathway mutations cannot be explained by mutator phenotypes

Two main acquired mutator phenotypes are well described in colorectal cancers, MSI caused by defective DNA mismatch repair and aberrant polymerase proofreading caused by *POLE* mutations. Both of these cancer types have a large excess of base substitutions. Based on previous models of “polygenic” tumorigenesis in hypermutant cancers (39), we wondered whether the multiple *BRAF* and Ras pathway mutations in class 2 and 3 *BRAF-*mutant colorectal cancers were driven by a mutator phenotype. Contrary to this hypothesis, in samples with available MSI data (Table 1), we found an overrepresentation of MSI in class 1–mutant tumors – reflecting the well-established MSI–V600E association (40) – with all class 2 and 3 tumors being MSI-negative (52.4% vs. 0%; *P* < 0.001, Fisher exact test). None of the class 2 and 3 *BRAF*-mutant colorectal cancers had pathogenic *POLE* mutations.

Class 1 *BRAF* mutations are also associated with the CpG Island Methylator Phenotype (CIMP) and with Wnt pathway activation via *RNF43* or *CTNNB1* mutations. Within the S:CORT dataset, class 1 colorectal cancers were predominantly CIMP-high (71/118, 60.2%), compared with only 26.7% (8/30, 26.7%) in class 3; (*P* = 0.002, Fisher exact test). The largest CIMP cluster for class 3–mutant colorectal cancers was CIMP-low at 40% (12/31), not significantly different from class 1 (27.1%, 32/118; *P* = 0.30).

Relatedly, 20.1% (133/661) of tumors with available data harbored a pathogenic *APC* mutation (12), whereas 81.4% (22/27) of class 2 and 76.7% (56/73) of class 3, respectively, harbored a pathogenic *APC* mutation (*P* < 0.001, Fisher exact tests.). We further found significantly lower frequencies of pathogenic *CTNNB1* mutation in class 2 and 3 tumors versus class 1 (1.02% and 1/98 vs. 16.7%, 96/575; *P* < 0.001, Fisher exact test), along with reduced frequencies of pathogenic *RNF43* mutation for class 2 and 3 versus class 1 tumors (0%, 0/98 vs. 22.9%, 147/643; *P* < 0.001, Fisher exact test). Thus, atypical *BRAF* mutations do not seem to follow the serrated/MSI/CIMP/RNF43 pathway of colorectal tumorigenesis but instead resemble cancers following the canonical pathway of colorectal tumorigenesis (12, 41).

### *BRAF* ^V600E^ mutations are under strong selective constraints, whereas atypical *BRAF* variants are subject to weaker constraints

Although we found no evidence that atypical *BRAF* mutations are associated with the MSI or *POLE* mutator phenotypes, it remained possible that their occurrence was driven by other mutational processes that are active in tumor cells or their precursors, mostly in the distal colorectum. We therefore generated whole-genome 96-channel (3nt context) mutational spectra for each of the 100KGP Genomics England tumors (WGS) data. The mean proportional activity of the class 1 (V600E) mutation channel, GTG>GAG, was 0.69%, compared with a summed average of 12.9% for the 16 class 2 and 3 mutation channels. The presence of a V600E mutation was not associated with activity of the causal channel (*P* = 0.286, logistic regression), whereas the presence of an atypical *BRAF* mutation was associated with higher activity of the causal channels (*P* = 0.028; Supplementary Table S6). These data are consistent with V600E being under strong selective constraints in the proximal large bowel whereas atypical *BRAF* mutations have weaker constraints and are influenced more by mutational processes.

### The CMSs of class 3–mutant *BRAF* colorectal cancers differ from those of class 1 mutants

The CMSs of colorectal cancer (42) represent a gene expression–based stratification into four subtypes. CMS1 (referred to as the *MSI Immune* subtype) is the predominant signature associated with class 1 *BRAF* mutations and is characterized by MSI, high CIMP, and hypermutation. Based on S:CORT data, we found a significant difference in CMS classification across all three *BRAF* mutation classes. CMS data were available for 114 class 1, 4 class 2, and 28 class 3 *BRAF-*mutant cancers. CMS1 predominated in class 1–mutant cancers, as expected (43). However, class 2 and class 3 tumors had a lower proportion of CMS1 and higher proportion of CMS2 (Fig. 3B; *P* < 0.001, Fisher exact tests). CMS3 and CMS4 occurred at similarly low frequencies in the three classes. CMS2 is generally regarded as the *canonical* CMS subtype, characterized by high levels of somatic CNAs and increased of Wnt and Myc signaling. To ensure that the apparent differences in CMS between *BRAF* mutation classes were not readily explained by potentially confounding variables, including MSI status and tumor location (see Materials and Methods), we further performed multiple logistic regression analyses (Supplementary Table S7). *BRAF* class 1–mutant colorectal cancers were significantly more likely to be CMS1 (OR = 6.68; *P* = 0.024) and significantly less likely to be CMS2 (OR = 0.01; *P* = 0.01) than cancers in the other two *BRAF* classes. Class 3–mutant colorectal cancers showed the opposite associations to class 1 and were significantly less likely to be CMS1 (OR = 0.15; *P* = 0.05) and highly likely to be CMS2 (OR = 33.04; *P* = 0.004). Class 2–mutant colorectal cancers were not associated with any significant differences in CMS versus non–class 2 *BRAF* cancers, representing an intermediate subgroup between classes 1 and 3. We additionally subtyped BRAF-mutant colorectal cancers using our own A-B-C positional classifier with consideration for additional Ras status (Supplementary Fig. S3).

### Greater EGFR ligand (*AREG/EREG*) expression in the distal colorectum may support a permissive environment for atypical *BRAF* mutations

*In vitro* evidence suggests that atypical *BRAF* mutations more weakly activate Ras/Raf–MEK–ERK signaling (2, 3). We hypothesized that although additional Ras pathway mutations are also likely to be weaker in their effects, they may be compensated for by another unknown source; for instance, there may exist a more permissive environment in the normal distal colorectum relative to the proximal colorectum, which might permit the emergence of weaker drivers such as class 3 *BRAF* mutations. Using normal bowel mRNA-seq data collected as part of the study by Fernandez-Rozadilla and colleagues (32), subsequently referred to as “INTERMPHEN,” we first examined the expression of core Ras pathway genes *KRAS*, *BRAF*, and *NRAS* in samples collected from the proximal colorectum (*n* = 119) and two sites in the distal colorectum (distal colon and rectum, *n* = 236). *KRAS* and *BRAF* pathway genes showed only modest differences in expression among site [*KRAS* log_2_ fold change (FC) = 0.12 and *BRAF* log_2_FC = −0.13; proximal vs. distal/rectal; *P*.adj < 0.001], whereas *NRAS* expression was more greatly underexpressed in the distal/rectal versus proximal colon (log_2_FC = −0.35; *P*.adj < 0.001). Replication across TCGA COADREAD normal tissue mRNA-seq data (*n* = 18 proximal and *n* = 19 distal/rectal), however, found no significant differences in *KRAS*, *BRAF*, *or NRAS* expression (Supplementary Table S8).

**Figure 5.**
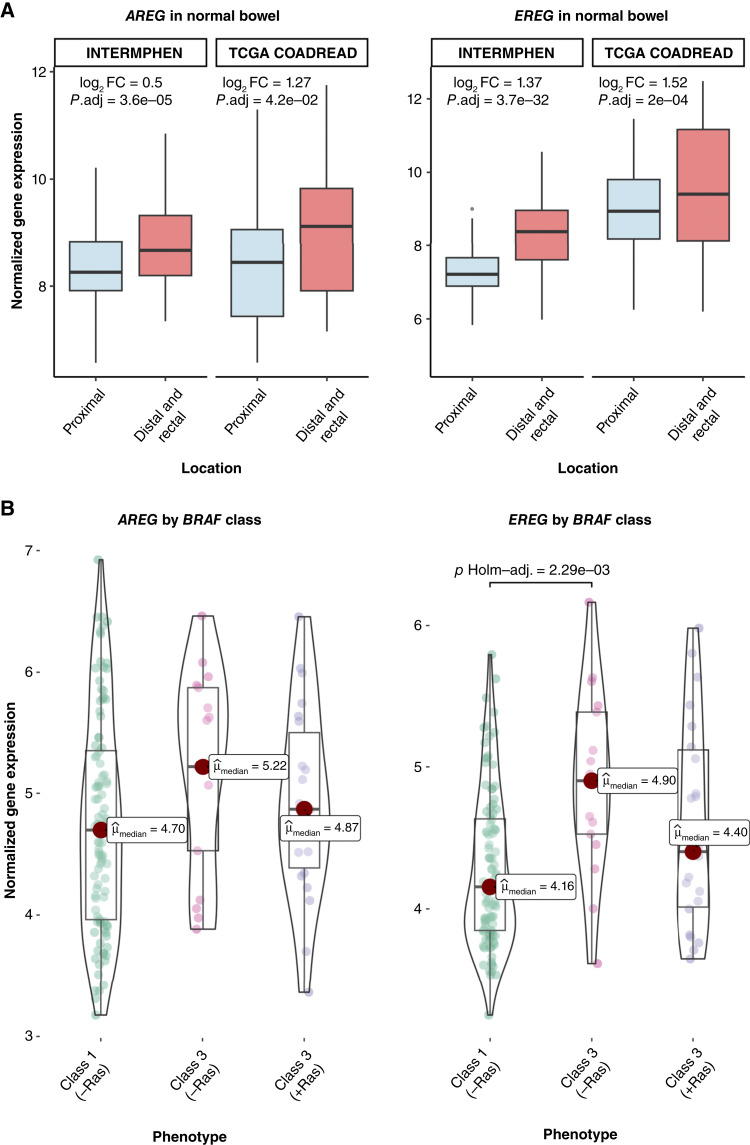
mRNA expression of the EGFR ligands *AREG* and *EREG*. **A,** Expression of *AREG* and *EREG* is significantly increased in the distal colorectum vs. proximal colon across the INTERMPHEN (*n* = 119 proximal and *n* = 236 distal colorectum) and TCGA COADREAD (*n* = 18 proximal and *n* = 19 distal/rectal). Log_2_FC and significance values obtained via DESeq2 analysis (see Materials and Methods). **B,** Expression of *EREG* is significantly increased in class 3 *BRAF*-mutant colorectal cancers vs. class 1 in the absence of a concomitant Ras mutation (*P*.adj < 0.005), which may reflect the distal colorectum bias of class 3 mutations. (Created with BioRender.com.)

Notably, we found that the EGFR ligands *AREG* or *EREG*, which may stimulate the Ras/Raf–MEK–ERK pathway via ligand drive (44, 45), showed significant regional differences in both the INTERMPHEN and TCGA normal cohorts. In the INTERMPHEN cohort, *EREG* expression was particularly elevated in the distal colon and rectum versus proximal colon (log_2_FC = 1.37; *P*.adj < 0.001), with *AREG* showing a similar but more modest pattern (log_2_FC = 0.50; *P*.adj < 0.001); this finding was consistent following independent replication in the TCGA COADREAD normal mRNA-seq cohort, with distal/rectal enrichment of *EREG* (log_2_FC = 1.52; *P*.adj < 0.01) and *AREG* (log_2_FC = 1.27; *P*.adj < 0.05) versus that in the proximal colon (Fig. 5A; Supplementary Table S8). Consistent with these regional differences in normal tissues, analysis of the S:CORT cohort, which contained sufficient atypical colorectal cancers with RNA data, showed significantly higher expression of *EREG* in class 3 *BRAF*-mutant tumors lacking additional Ras mutations compared with class 1 mutants (*P* < 0.005, Dunn test; Fig. 5B). Although this difference in *EREG* expression between *BRAF* classes may partially reflect their distinct anatomic distributions, the observation that class 3 *BRAF* mutants with additional Ras pathway mutations showed similar EGFR ligand expression to class 1 mutants suggests that elevated *EGFR* ligand expression might not be necessary when additional Ras pathway mutations are present. These findings suggest a natural gradient of EGFR ligand expression along the colorectum which may indeed contribute to the regional distribution of *BRAF* mutation classes, potentially providing a more supportive environment for weaker oncogenic drivers in the distal colon and rectum.

### Raf signaling levels are highest in class 1 *BRAF*-mutant cancers, whereas class 3 *BRAF* mutants increase Raf signaling by additional Ras pathway mutations

To explore the *BRAF*-mutant transcriptome further and to identify other differences between tumors with typical and atypical *BRAF*, we performed a hypothesis-free search for differential gene set expression between class 3–mutant and class 1–mutant tumors from S:CORT using GSEA based on C6 oncogenic gene sets. As baseline, we first compared class 3 mutants with class 1, in both cases without additional Ras mutations. Of nine significantly enriched (*q* < 0.05) transcriptome-wide gene sets (Supplementary Table S9A), at least three were clearly associated with higher Ras/Raf–MEK–ERK signaling in the class 1 tumors: *ERRB2_UP*.*V1* (*q* = 0.005), *MEK_UP*.*V1* (*q* = 0.007), and *EGFR_UP.V1* (*q* = 0.032). Furthermore, another gene set related to Wnt decreased *MYC* signaling and was lower in class 1 than in class 3 (MYC_UP.V1_DN, *q* = 0.042). Comparisons between class 1 (lacking extra Ras) and class 3 colorectal cancers with additional Ras (either typical or atypical) seemed to eliminate significant differences between subtypes, with none of the above gene sets emerging at *q* < 0.05 (Supplementary Table S9B). Comparing class 3 *BRAF* lacking additional Ras with class 3 with typical Ras mutations showed higher SINGH_KRAS_DEPENDENCY_SIGNATURE (*q* = 0.032) and *ERBB2_UP.V1* gene set expression (*q* = 0.043; Supplementary Table S9C) in the latter. However, comparison of class 3 *BRAF* lacking additional Ras with class 3 with additional atypical Ras yielded no significant gene sets at *q* < 0.05 (Supplementary Table S9D). Finally, no significant differences were found between the relatively few class 3 mutants harboring atypical and typical Ras (Supplementary Table S9E).

### Associations between atypical *BRAF* mutations and prognosis

Previous work has proposed that non-*BRAF*^V600E^–mutant colorectal cancers have a relatively good prognosis (5–7, 35), although not all such analyses have controlled for potential confounders such as MSI and tumor location or taken into account additional Ras pathway mutations. We therefore investigated OS in the six cohorts with available data (Supplementary Material). An initial uncorrected log-rank analysis showed a borderline significantly better OS for class 3 *BRAF* mutations versus class 1, provided that both lacked additional Ras mutations, in agreement with previous studies (*P* = 0.058, log-rank test, Supplementary Fig. S4A).

Owing to the combination of multiple patient cohorts in our dataset and the strong possibility of confounders, we proceeded to a more robust approach utilizing mixed-effects Cox proportional hazards models, in which study was treated as a random variable, whereas patient characteristics were treated as fixed-effects variables (see Materials and Methods). Initially, we confirmed longer OS for class 3 than for class 1 *BRAF*-mutant colorectal cancers without concomitant pathogenic Ras pathway mutations (HR = 0.25; *P* = 0.011). No significant difference in survival was observed for class 2 *BRAF* mutations relative to class 1 (HR = 1.13; *P* = 0.85; Table 2, model I).

**Table 2.** Survival analysis of *BRAF*-mutant colorectal cancer classes: multivariate and univariate mixed-effects Cox proportional hazards models. Model I compares *BRAF* classes across all patients irrespective of additional Ras mutation; model II compares class 1 without Ras vs. class 3 with Ras mutations; model III examines additional Ras mutation status within class 3. All models include study as a random effect. Both univariate and multivariate analyses were performed on identical patient cohorts per model to ensure direct comparability.

	Model I				Model II				Model III			
Sample size	247				238				27			
Events	90				93				14			
Variable	Multivariate HR (95% CI)	*P*	Univariate HR (95% CI)	*P*	Multivariate HR (95% CI)	*P*	Univariate HR (95% CI)	*P*	Multivariate HR (95% CI)	*P*	Univariate HR (95% CI)	*P*
BRAF classification												
Class 1	(Reference)	—	(Reference)	—	(Reference)	—	(Reference)	—	^a^	—	^a^	—
Class 2	1.13 (0.32–3.94)	0.85	1.36 (0.42–4.41)	0.61	—	—	—	—	^a^	—	^a^	—
Class 3	0.25 (0.08–0.73)	0.01	0.4 (0.13–1.21)	0.11	0.94 (0.46–1.91)	0.86	1.09 (0.54–2.19)	0.8	^a^	—	^a^	—
Clinical features												
Age at diagnosis	1.01 (0.98–1.03)	0.68	0.99 (0.96–1.01)	0.22	1.00 (0.98–1.02)	0.99	0.98 (0.96–1)	0.12	1.04 (0.93–1.16)	0.52	0.97 (0.91–1.03)	0.29
Sex (female)	(Reference)	—	(Reference)	—	(Reference)	—	(Reference)	—	(Reference)	—	(Reference)	—
Sex (male)	1.30 (0.82–2.07)	0.27	1.15 (0.73–1.80)	0.56	1.24 (0.79–1.97)	0.35	1.16 (0.75–1.81)	0.5	1.67 (0.20–14.1)	0.48	2.61 (0.68–10.1)	0.16
Left-sided	(Reference)	—	(Reference)	—	(Reference)	—	(Reference)	—	(Reference)	—	(Reference)	—
Right-sided	0.97 (0.51–1.87)	0.93	1.39 (0.75–2.57)	0.3	1.06 (0.56–2.03)	0.85	1.28 (0.68–2.40)	0.44	0.60 (0.14–2.50)	0.64	0.73 (0.23–2.31)	0.59
Disease staging												
Stage I	(Reference)	—	(Reference)	—	(Reference)	—	(Reference)	—	(Reference)	—	(Reference)	—
Stage II	1.32 (0.29–5.93)	0.72	1.32 (0.30–5.82)	0.72	2.44 (0.32–18.96)	0.39	2.41 (0.31–18.6)	0.4	^b^	—	^b^	—
Stage III	2.91 (0.69–12.38)	0.15	2.47 (0.59–10.4)	0.22	5.21 (0.71–38.3)	0.11	5.41 (0.74–39.7)	0.1	^b^	—	^b^	—
Stage IV	16.6 (3.78–73.2)	<0.001	14.2 (3.31–61)	<0.001	28 (3.77–207.5)	<0.001	35.2 (4.8–256)	<0.001	^b^	—	^b^	—
Additional features												
MSI	(Reference)	—	(Reference)	—	(Reference)	—	(Reference)	—	(Reference)	—	(Reference)	—
MSS	1.39 (0.80–2.42)	0.25	1.12 (0.63–2)	0.69	1.41 (0.81–2.44)	0.23	1.46 (0.81–2.62)	0.21	^b^	—	^b^	—
Additional Ras status												
No additional Ras	^a^	—	^a^	—	^a^	—	^a^	—	(Reference)	—	(Reference)	—
Additional Ras	^a^	—	^a^	—	^a^	—	^a^	—	4.92 (1.12–21.6)	0.03	4.17 (1.16–14.9)	0.03

Abbreviation: CI, confidence interval.

a Variable not included in model specification.

b Variable could not be reliably estimated in model III because of small subgroups.

We subsequently explored whether class 3 cancers harboring additional Ras mutations had a significantly different prognosis from that of class 1 (all V600E). No difference in OS was detected between the two groups (class 3 plus Ras HR = 0.93; *P* = 0.86; Table 2, model II). As concomitant Ras pathway mutations seemed to make the survival of patients with class 3 colorectal cancer similar to that of class 1, we compared the OS of class 3 cancers with and without additional Ras pathway mutations. We detected significantly worse OS of the tumors with additional Ras mutations (HR = 4.92; *P* = 0.03; Table 2, model III). Overall, our results suggest that *BRAF* class 3 colorectal cancers have better survival than class 1 colorectal cancers, but the additional Ras pathway mutations in some class 3 BRAF-mutant colorectal cancers result in survival similar to that associated with class 1 V600E. For visualization purposes, unadjusted Kaplan–Meier curves representing the above survival comparisons are presented in Supplementary Fig. S4, whereas statistical results are derived from the mixed-effects models that account for potential confounders and study-specific effects.

### Determining whether atypical *KRAS* mutations are associated with additional Ras pathway mutations

By analogy with *BRAF* mutations, we wondered whether atypical *KRAS* mutations in colorectal cancers without *BRAF* mutations also tended to have concomitant mutations in Ras pathway genes (Supplementary Fig. S5). Of 335 colorectal cancers harboring atypical *KRAS* mutations in the combined cohorts, 317 (94.6%) had no concomitant pathogenic *BRAF* mutation, with *BRAF* mutations in the remaining 18 all belonging to class 3 (18/335, 5.4%). Of these 317 cases, 13 (4.1%) harbored an additional pathogenic Ras pathway mutation (analysis restricted to *NRAS* and *NF1*). Of the 335 colorectal cancers, 13 (3.88%) with atypical *KRAS* also carried a typical *KRAS* mutation, and none of those tumors had an additional *BRAF*, *NRAS*, or *NF1* mutation. Most cancers with atypical *KRAS* mutations (291/335, 86.9%) had no additional mutations in *BRAF*, *NRAS*, *or NF1*, with the incidence of these mutations being far lower than the incidence of concomitant Ras mutations in atypical *BRAF* colorectal cancers (Table 1). It seems, therefore, that atypical *KRAS* mutations may generally cause sufficient Ras dysregulation to drive tumorigenesis.

## Discussion

We set out to obtain clues about why some colorectal cancers grow with atypical *BRAF* driver mutations that are predicted to have impaired or suboptimal kinase activity and thus result in weak downstream MAPK signaling in comparison with the most frequently observed V600E class 1 variant. We have shown that atypical (class 2 or 3) *BRAF* mutations are associated with the presence of additional mutations in the Ras pathway. These frequently take the form of pathogenic changes at sites other than the *KRAS* codon 12, 13, and 61 hotspots. The additional Ras mutations probably have relatively weak effects on Ras activation (46) and seem to be present in the same cells as the atypical *BRAF* changes rather than in a separate subclone. These associations seem stronger for class 3 (loss-of-function) mutations than for class 2 (active dimer) mutations, consistent with a model in which kinase-dead class 3 *BRAF* mutants require concomitant Ras activation to be pathogenic (3). In class 3 cancers that do not have additional Ras pathway mutations, this model predicts that other sources of Ras activation may be present. In support of the model, we found the evidence of higher EGFR ligand expression in class 3 than in class 1 tumors, although we cannot exclude the possibility that this results from the effects of feedback inhibition. Importantly, in normal bowel RNA-seq data, we identified increased basal *AREG* and *EREG* expression in the distal versus proximal colorectum. This regional variation in EGFR signaling may contribute to the observed class 3 versus class 1 differences and could explain why the distal colorectum provides a more permissive environment for atypical *BRAF* mutations to drive tumorigenesis. However, we note that transcriptomic data alone cannot fully capture pathway regulation at the protein level.

Class 2 mutations seem to be intermediate between classes 1 and 3 in most respects. For example, colorectal cancers with class 1 *BRAF* mutations are likely to be CMS1, whereas class 3 mutants are rarely CMS1 and class 2 mutants fall into either group. Relating *BRAF* mutation types to underlying mutational signatures, it is likely that class 1 *BRAF* mutants are strongly selected (as V600E changes are not commonly created) and that class 2 or 3 mutants are under weaker selective constraints in the distal colorectum, where they are overrepresented.

As class 3 mutations of codon 466 seem to be outliers (e.g., usually located proximally), we explored an “ABC” classifier based on functional domains within BRAF (Supplementary Material). Associations similar in strength to the “class 1-2-3” groups were found. It is possible that the classification of *BRAF* driver mutations will be refined over time and, we speculate, that a further group, principally comprising codon 466 changes, will emerge.

The combination of atypical *BRAF* and other atypical Ras mutations (in *KRAS*, *NRAS*, or *NF1*) is consistent with a “mini-driver” model in which some cancers acquire sufficient Ras activation through a two-step process rather than a single “hit” at *BRAF*^V600E^ or one of the common *KRAS* hotspots. In support of this suggestion, class 2 and 3 changes seem to be favored over class 1 by the mutational processes active in the cancer cells. Our cohorts are insufficient to determine the order of acquisition of *BRAF* and additional Ras mutations. We further explored whether atypical *KRAS* mutations presented with concomitant Ras/Raf at frequencies similar to those of atypical *BRAF*, but the former was significantly less frequent, perhaps reflecting the fact that atypical *KRAS* mutants possess some increased kinase activity in contrast to the loss of function associated with class 3 *BRAF* mutations.

In an attempt to mitigate potentially confounding variables present in previous studies, we used mixed-effects Cox proportional hazards models accounting for covariates (e.g., age, sex, tumor stage, MSI subtype, location, and study) to analyze survival. Consistent with previous work (5–7, 35), we observed better OS for patients with a class 3 *BRAF* mutation versus class 1 (V600E). Our data suggest, however, that this benefit may be reduced, or absent, for cancers carrying both atypical *BRAF* and additional Ras mutations. Previously, Yaeger and colleagues (1) had performed a retrospective, multicenter study to characterize the response of non-*BRAF*^V600E^ variants to a range of anti-EGFR treatment regimens, querying datasets from five centers for patients with metastatic colorectal cancer with non-*BRAF*^V600E^ between 2010 and 2017. They found 8% and 50% response rates for class 2 and 3 mutations, respectively. A possible rationale for this difference in response rates is that the inhibition of EGFR leads to a reduction in Ras signaling and thus inhibition of kinase-dead class 3 *BRAF* mutants, which still require phosphorylation by Ras-GTP. Class 2–mutant *BRAF*, however, functions as Ras-independent homodimers, and thus the inhibition of upstream Ras would have little effect. As little under half of *BRAF* class 3 colorectal cancers in our analysis carry additional pathogenic Ras pathway mutations, we speculate that the efficacy of anti-EGFR therapy in this subset of double-mutant patients may be diminished.

Our work levers a large, combined dataset in conjunction with a comprehensive molecular evaluation of relevant Ras pathway genes, as opposed to focusing upon primarily hotspot mutations. The use of multiple datasets is almost unavoidable for studying rare driver mutation genotypes and hence providing precision medicine for the patients concerned. We recognize, nonetheless, that our study cohorts may be heterogeneous in their methods, samples, inclusion criteria, and data collected. There is certainly an argument for large clinicopathologic–molecular studies of patients with colorectal cancer with class 2 or 3 *BRAF* mutations.

In summary, our findings shed further light on the classes of atypical *BRAF* mutations in colorectal cancer, which differ both molecularly and pathologically from typical *BRAF*-mutant disease. We believe these findings highlight the importance of whole-gene sequencing of patient samples in the clinic, with many atypical (and likely actionable) pathogenic mutations falling outside routinely assayed hotspot regions. With non-*BRAF*^V600E^–mutant colorectal cancers gaining interest due to the apparent positive prognosis, we hope that our work will stimulate clinical consideration of the potential confounding effects of additional, often noncanonical, Ras pathway mutations in this subset of disease.

## Supplementary Material

Supplementary Figure 1 Supplementary Figure 1. Study and data availability overview (see Supplementary Table 1).

Supplementary Figure 2 Supplementary Figure 2. Scaled Schoenfeld residuals for corresponding fixed-effect models, inclusive of study as a categorical variable, relating to mixed-effects survival models in Table 2. A. Model I compares BRAF classes across all patients irrespective of additional Ras mutation; B. Model II compares Class 1 without Ras vs Class 3 with Ras mutations; C. Model III examines additional Ras mutation status within Class 3. P-values represent significance of violation of proportional hazards assumptions.

Supplementary Figure 3 Supplementary Figure 3. Molecular subtyping of BRAF-mutant CRCs into the consensus molecular subtypes of CRC (CMS). Left: CMS groupings within BRAF classes 1 to 3. Right: CMS groups when stratified with the A-B-C positional classifier.

Supplementary Figure 4 Supplementary Figure 4. Kaplan-Meier survival curves of unadjusted overall survival (OS, months) for CRCs harboring a Class 1, 2, or 3 BRAF mutation. A: Overall survival by BRAF class irrespective of additional Ras status. B: Class 1, 2, and 3 in the absence of additional Ras. C: Class 1 and 3 absent additional Ras, and class 3 with additional Ras. Survival as shown is not corrected for covariates, whereas mixed-effects models including several covariates are reported in the main text and Table 2. Class 2 samples with survival data containing additional Ras status were insufficient for this analysis, as discussed in the main text.

Supplementary Figure 5 Supplementary Figure 5. Atypical KRAS mutations and additional Ras pathway mutations. A CONSORT-style diagram shows the breakdown of tumours with atypical KRAS mutations by accompanying mutations in KRAS, BRAF, NRAS or NF1.

Supplementary Text 1 Supplementary Text 1. Comparison between the A-B-C and 1-2-3 BRAF classification systems.

Supplementary Table 1 Supplementary Table 1. Details of each cohort included in the study

Supplementary Table 2 Supplementary Table 2. PCR Primer sequences for validation of FFPE samples

Supplementary Table 3 Suplementary Table 3a-b. Summarised BRAF mutation characteristics per cohort following the 1-2-3 classification system (Table 3a) and the A-B-C classification system (Table 3b). Table cells with values less than 5 have been censored to facilitate Genomics England reporting restrictions. An uncensored version of Supplementary Table 3 is available within the Genomics England 100,000 Genomes Project Research Environment upon request.

Supplementary Table 4 Supplementary Table 4. Summary data reclassified using a positional based system based on proximity to functional domains, which may hold value in exploring atypical BRAF variants not yet functionally assessed (Classes A, B, and C). A single instance of co-occurring class 2 and 3 BRAF is omitted from the total counts yet presented as MSS with no additional clinical data available.

Supplementary Table 5 Supplementary Table 5. 100,000 Genomes Project: Clonality estimates for atypical variants harbouring additional Ras pathway mutation with sufficient data available.

Supplementary Table 6 Supplementary Table 6. Logistic regression model of atypical BRAF mutation versus V600E status with covariates MSI status, age, sex, and tumour location.

Supplementary Table 7 Supplementary Table 7. Logistic regression models of CMS groups by BRAF mutation class, with covariates: tumour location (left or right colon), MSI status, age, sex, and additional Ras status (Class 1/2/3 left, A-B-C right).

Supplementary Table 8 Supplementary Table 8. Differential gene expression results (post-DESeq2 analysis) for BRAF, KRAS, NRAS, and the EGFR-ligands AREG and EREG across two normal-colorectum RNAseq cohorts. Sorted by adjusted p.values (Benjamini-Hochberg).

Supplementary Table 9 Supplementary Tables 9a-c. Gene-set enrichment analaysis (GSEA) results comparing classes of BRAF-mutant CRCs with and without additional Ras mutation.

Supplementary Data 1 Supplementary Data 1. Sanger sequencing chromatograms presented in Figure 3C-D.
