# Supplementary material for: Coevolution of Atypical BRAF and KRAS Mutations in Colorectal Tumorigenesis
Source: Mol Cancer Res. 2025 Jan 3;23(4):300–12. doi: 10.1158/1541-7786.MCR-24-0464 (PMC7617415; doi:10.1158/1541-7786.MCR-24-0464)
Supplement: Supplementary Figure 1 — Study and data availability overview (see Supplementary Table 1). [file mcr-24-0464_supplementary_figure_1_suppsf1.pptx]

## Slide 1
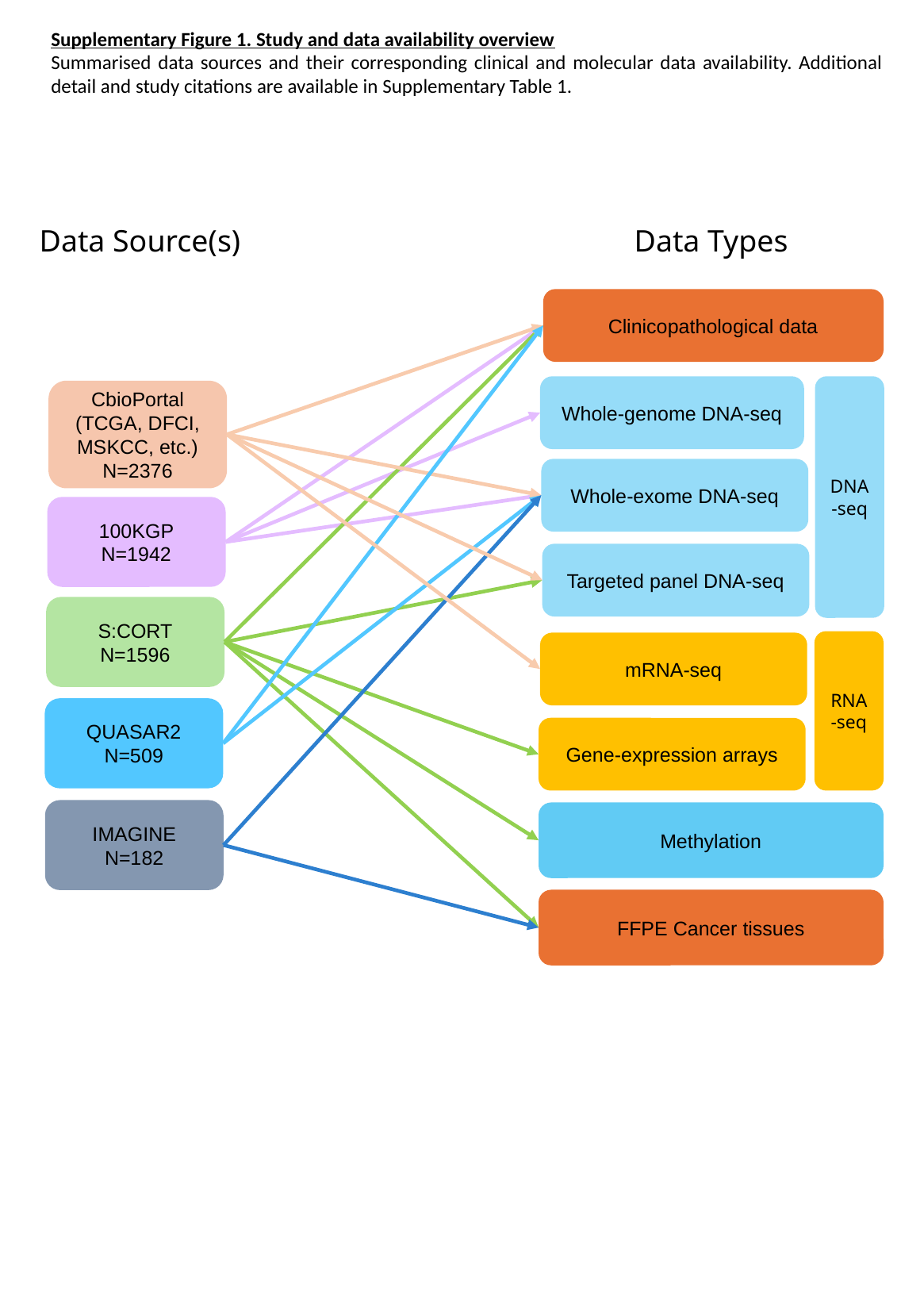

Supplementary Figure 1. Study and data availability overview
Summarised data sources and their corresponding clinical and molecular data availability. Additional detail and study citations are available in Supplementary Table 1.
Data Types
Data Source(s)
Clinicopathological data
Whole-genome DNA-seq
CbioPortal (TCGA, DFCI, MSKCC, etc.)
N=2376
Whole-exome DNA-seq
100KGP
N=1942
S:CORT
N=1596
mRNA-seq
QUASAR2
N=509
IMAGINE
N=182
Methylation
FFPE Cancer tissues
DNA -seq
Targeted panel DNA-seq
RNA-seq
Gene-expression arrays
