# Supplementary material for: Coevolution of Atypical BRAF and KRAS Mutations in Colorectal Tumorigenesis
Source: Mol Cancer Res. 2025 Jan 3;23(4):300–12. doi: 10.1158/1541-7786.MCR-24-0464 (PMC7617415; doi:10.1158/1541-7786.MCR-24-0464)
Supplement: Supplementary Figure 2 — Scaled Schoenfeld residuals for corresponding fixed-effect models, inclusive of study as a categorical variable, relating to mixed-effects survival models in Table 2. A. Model I compares BRAF classes across all patients irrespective of additional Ras mutation; B. Model II compares Class 1 without Ras vs Class 3 with Ras mutations; C. Model III examines additional Ras mutation status within Class 3. P-values represent significance of violation of proportional hazards assumptions. [file mcr-24-0464_supplementary_figure_2_suppsf2.pptx]

## Slide 1
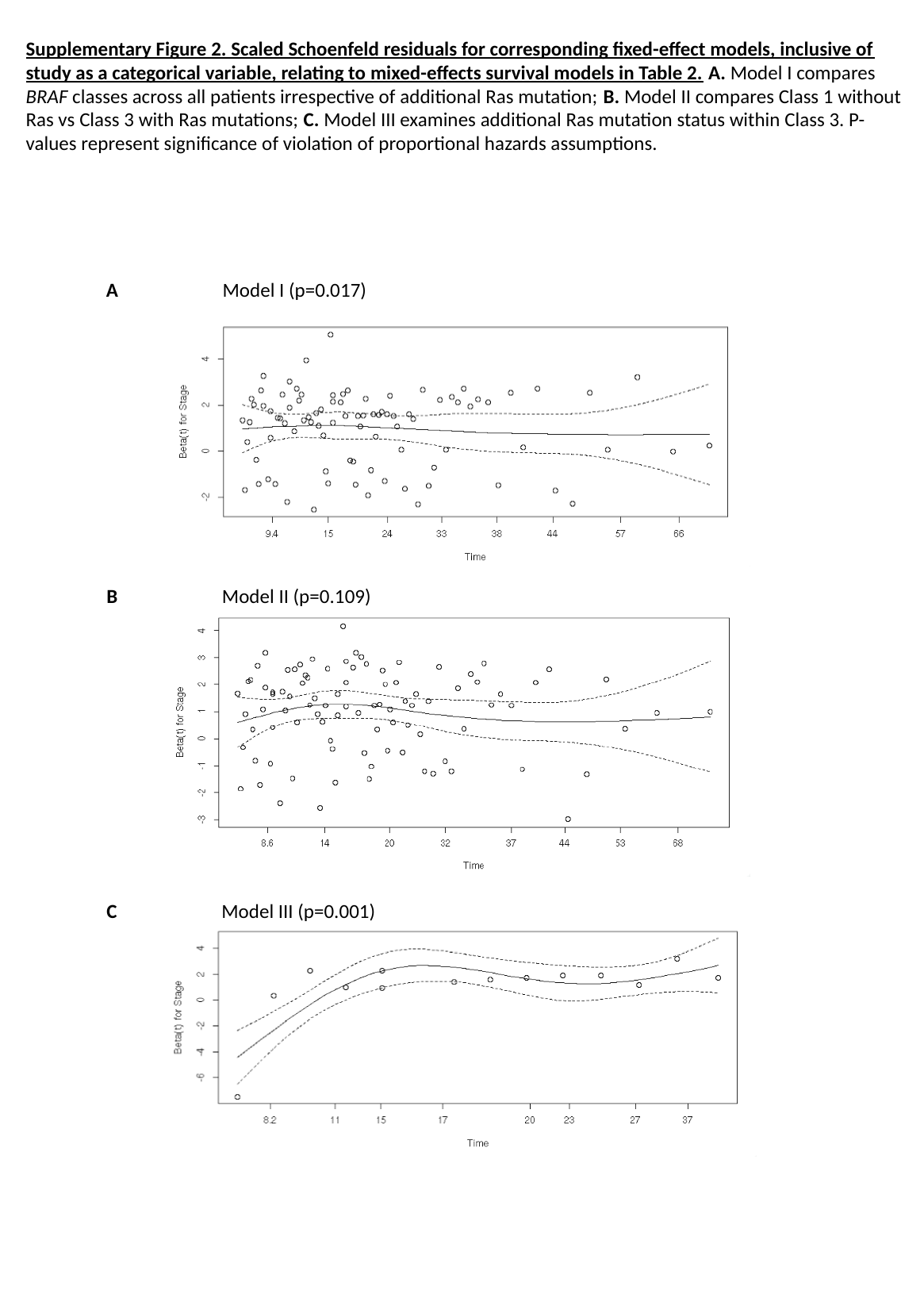

Supplementary Figure 2. Scaled Schoenfeld residuals for corresponding fixed-effect models, inclusive of study as a categorical variable, relating to mixed-effects survival models in Table 2. A. Model I compares BRAF classes across all patients irrespective of additional Ras mutation; B. Model II compares Class 1 without Ras vs Class 3 with Ras mutations; C. Model III examines additional Ras mutation status within Class 3. P-values represent significance of violation of proportional hazards assumptions.
A Model I (p=0.017)
B Model II (p=0.109)
C Model III (p=0.001)
