# Supplementary material for: Coevolution of Atypical BRAF and KRAS Mutations in Colorectal Tumorigenesis
Source: Mol Cancer Res. 2025 Jan 3;23(4):300–12. doi: 10.1158/1541-7786.MCR-24-0464 (PMC7617415; doi:10.1158/1541-7786.MCR-24-0464)
Supplement: Supplementary Figure 3 — Molecular subtyping of BRAF-mutant CRCs into the consensus molecular subtypes of CRC (CMS). Left: CMS groupings within BRAF classes 1 to 3. Right: CMS groups when stratified with the A-B-C positional classifier. [file mcr-24-0464_supplementary_figure_3_suppsf3.pptx]

## Slide 1
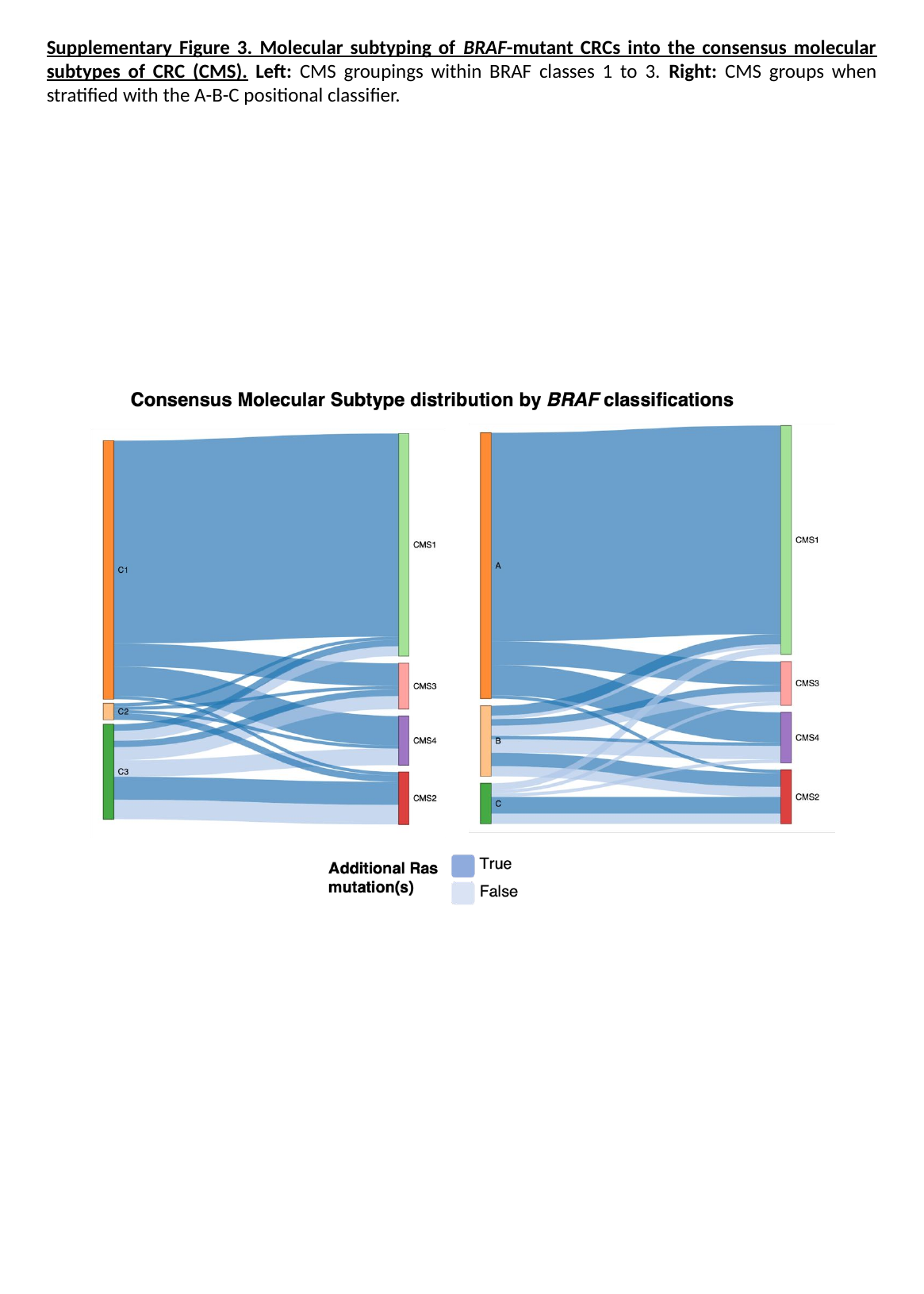

Supplementary Figure 3. Molecular subtyping of BRAF-mutant CRCs into the consensus molecular subtypes of CRC (CMS). Left: CMS groupings within BRAF classes 1 to 3. Right: CMS groups when stratified with the A-B-C positional classifier.
