# Supplementary figures and images for: Coevolution of Atypical BRAF and KRAS Mutations in Colorectal Tumorigenesis
Source: Mol Cancer Res. 2025 Jan 3;23(4):300–12. doi: 10.1158/1541-7786.MCR-24-0464 (PMC7617415; doi:10.1158/1541-7786.MCR-24-0464)

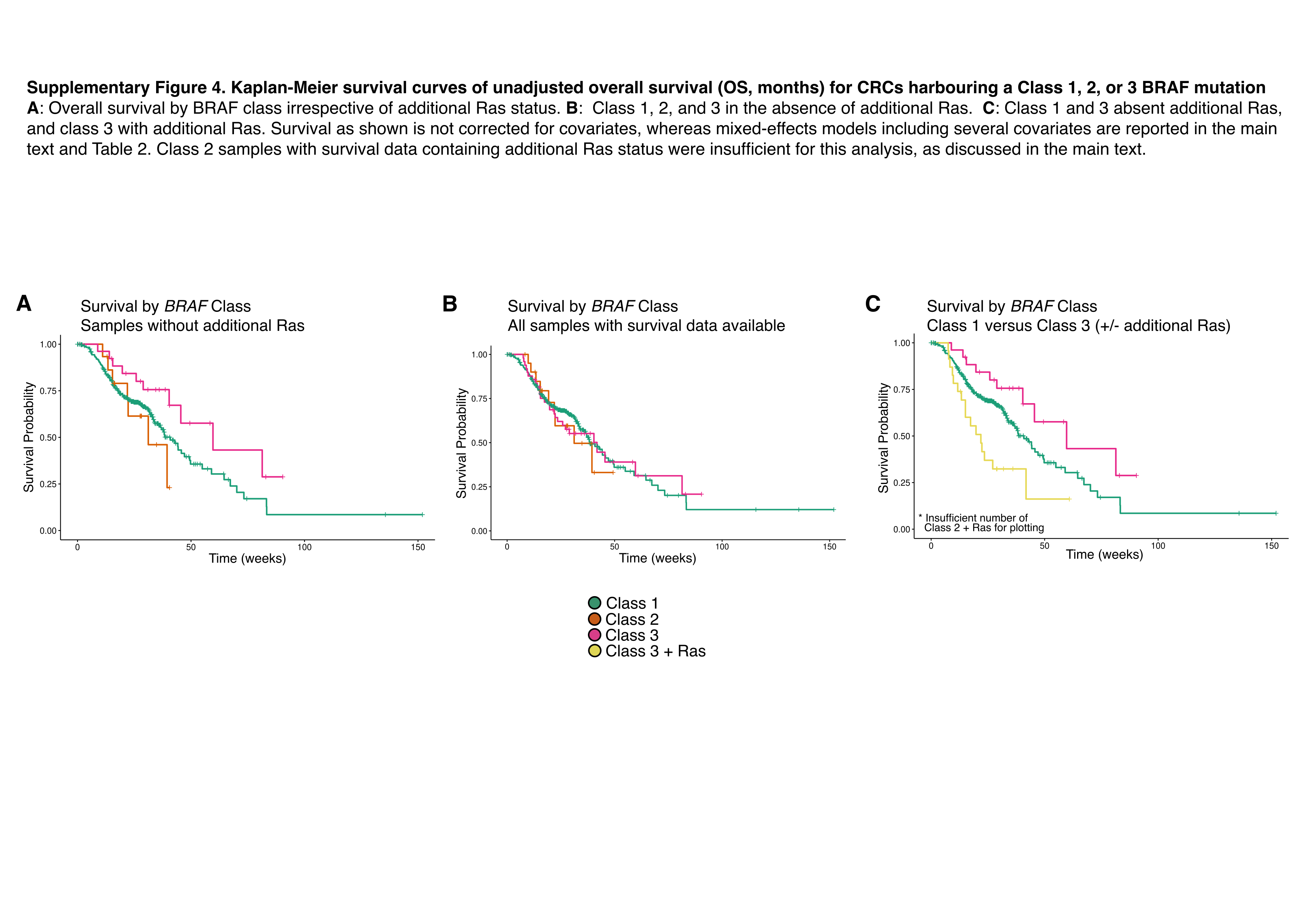

Supplement: Supplementary Figure 4 — Kaplan-Meier survival curves of unadjusted overall survival (OS, months) for CRCs harboring a Class 1, 2, or 3 BRAF mutation. A: Overall survival by BRAF class irrespective of additional Ras status. B: Class 1, 2, and 3 in the absence of additional Ras. C: Class 1 and 3 absent additional Ras, and class 3 with additional Ras. Survival as shown is not corrected for covariates, whereas mixed-effects models including several covariates are reported in the main text and Table 2. Class 2 samples with survival data containing additional Ras status were insufficient for this analysis, as discussed in the main text. [file mcr-24-0464_supplementary_figure_4_suppsf4.png]
