# Supplementary material for: Coevolution of Atypical BRAF and KRAS Mutations in Colorectal Tumorigenesis
Source: Mol Cancer Res. 2025 Jan 3;23(4):300–12. doi: 10.1158/1541-7786.MCR-24-0464 (PMC7617415; doi:10.1158/1541-7786.MCR-24-0464)
Supplement: Supplementary Figure 5 — Atypical KRAS mutations and additional Ras pathway mutations. A CONSORT-style diagram shows the breakdown of tumours with atypical KRAS mutations by accompanying mutations in KRAS, BRAF, NRAS or NF1. [file mcr-24-0464_supplementary_figure_5_suppsf5.pptx]

## Slide 1
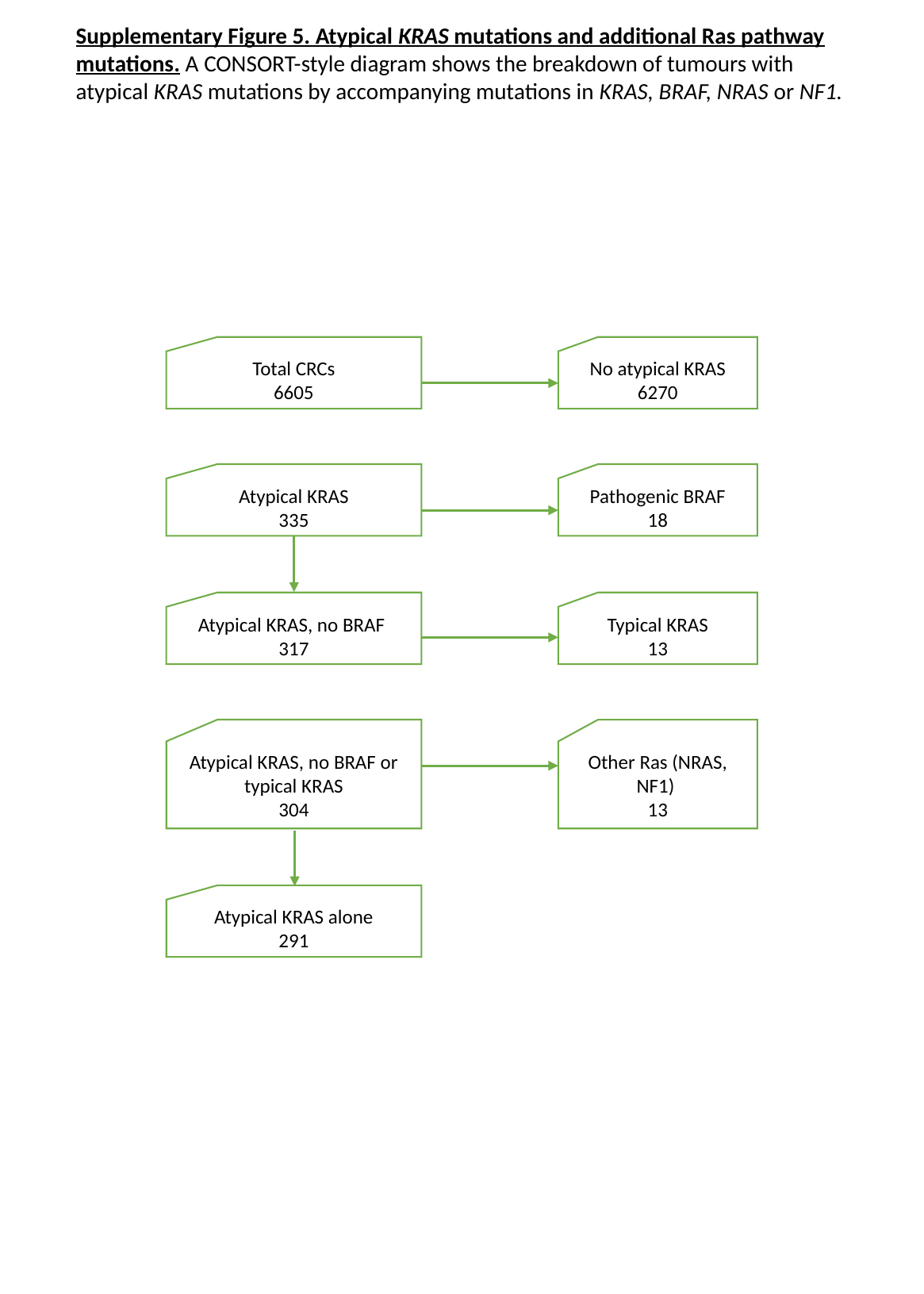

Supplementary Figure 5. Atypical KRAS mutations and additional Ras pathway mutations. A CONSORT-style diagram shows the breakdown of tumours with atypical KRAS mutations by accompanying mutations in KRAS, BRAF, NRAS or NF1.
Total CRCs
6605
No atypical KRAS
6270
Atypical KRAS
335
Pathogenic BRAF
18
Atypical KRAS, no BRAF
317
Typical KRAS
13
Atypical KRAS, no BRAF or typical KRAS
304
Other Ras (NRAS, NF1)
13
Atypical KRAS alone
291
