# Supplementary material for: Coevolution of Atypical BRAF and KRAS Mutations in Colorectal Tumorigenesis
Source: Mol Cancer Res. 2025 Jan 3;23(4):300–12. doi: 10.1158/1541-7786.MCR-24-0464 (PMC7617415; doi:10.1158/1541-7786.MCR-24-0464)
Supplement: Supplementary Text 1 — Comparison between the A-B-C and 1-2-3 BRAF classification systems. [file mcr-24-0464_supplementary_text_1_supps1.docx]

**Supplementary Text 1. Comparison between the A-B-C and 1-2-3 *BRAF* classification systems.**

The 1-2-3 *BRAF* mutation classification is based on *in vitro* assays of Ras pathway activity. However, as it has not been validated by analysis of native mutations in isogenic cells, we explored, in parallel, a simpler classification based on mutation location in functional domains which is presented alongside Figure 4.

Classes B and C tended to have additional Ras pathway mutations compared with class A (2.54% for class A versus 35.6% and 52.5% for B and C respectively, P<0.001, Fisher’s exact tests) (Supplementary Table 6). MSS was also significantly more frequent in classes B and C (52.4% MSI in class A versus 0% for classes B and C, p<0.001, Fisher’s exact tests). Noting that class 3 mutations at codon 466 were over-represented in the proximal colon compared with other class 3 variants, we found that group C variants as a whole (including codon 466) were associated with proximal location (70% group B vs 40.6% group C in distal colon and rectum, P<0.005, Fisher’s exact test; Supplementary Table 6).
